# An *In Silico* Insight into Novel Therapeutic Interaction of LTNF Peptide-LT10 and Design of Structure Based Peptidomimetics for Putative Anti-Diabetic Activity

**DOI:** 10.1371/journal.pone.0121860

**Published:** 2015-03-27

**Authors:** Sonali Gopichand Chavan, Deepti Dileep Deobagkar

**Affiliations:** 1 Bioinformatics Centre, University of Pune, Pune, Maharashtra, India; 2 Department of Zoology, Center of Advanced Studies, University of Pune, Pune, Maharashtra, India; Bioinformatics Institute, SINGAPORE

## Abstract

Lethal Toxin Neutralizing Factor (LTNF) obtained from Opossum serum (*Didephis virginiana*) is known to exhibit toxin-neutralizing activity for envenomation caused by animals, plants and bacteria. Small synthetic peptide- LT10 (10mer) derived from N-terminal fraction of LTNF exhibit similar anti-lethal and anti-allergic property. In our *in silico* study, we identified Insulin Degrading Enzyme (IDE) as a potential target of LT10 peptide followed by molecular docking and molecular dynamic (MD) simulation studies which revealed relatively stable interaction of LT10 peptide with IDE. Moreover, their detailed interaction analyses dictate IDE-inhibitory interactions of LT10 peptide. This prediction ofLT10 peptide as a novel putative IDE-inhibitor suggests its possible role in anti-diabetic treatment since IDE- inhibitors are known to assist treatment of Diabetes mellitus by enhancing insulin signalling. Furthermore, series of structure based peptidomimetics were designed from LT10 peptide and screened for their inhibitory interactions which ultimately led to a small set of peptidomimetic inhibitors of IDE. These peptidomimetic thus might provide a new class of IDE-inhibitors, those derived from LT10 peptide.

## Introduction

Lethal Toxin Neutralizing Factor (LTNF), an anti-lethal factor isolated from Opossum (*Didephisvirginiana*) serum is known to neutralize the lethality of venoms from all major snake families. The neutralizing effect of LTNF on venom of Cobra, Russell's Viper, rattlesnake, sea snake etc. along with scorpion venom, honey bee venom, plant derived ricin toxin and bacterial toxin botulinum have been experimentally shown by Lipps,B.V[1]. Thus, LTNF has potential as a universal therapy for envenomation[1–4]. Synthetic peptides with 10 amino acids designated as LT-10 (LKAMDPTPPL), derived from the N-terminal fraction of LTNF, could evoke similar lethal toxin neutralizing property as that of LTNF[2–4]. LTNF and LT-10 peptide inhibited lethality of animal, plant, and bacteria toxins when tested on mice without reacting to the nontoxic substances. Therefore, the use of LT10 peptide as an anti-allergic as well as broad-spectrum therapy for snake envenomation has been suggested[4–6]. Furthermore, LT10 peptide have been implicated to exhibit therapeutic role in asthma, diabetes mellitus, depression and autoimmune disease owing to its reported potential in reducing free IgE levels. LT10 treatment is believed to be ideal for these diseases and also has no observable side effects [6–8].

In our search for novel potential targets of LT10 peptide, we predicted few snake venom enzymes for their possible inhibition by LT10 peptide [9]. In present work, we identified Insulin Degrading Enzyme (IDE) as one such interacting partner and studied relatively stable and inhibitory interaction of LT10 peptide with IDE. Insulin degrading enzyme, also referred to as Insulysin, insulin protease or insulinase is an evolutionarily conserved zinc metallopeptidase found in bacteria, fungi, plants and animals[10, 11]. IDE is believed to be a primary regulator of proteolytic degradation and inactivation of insulin[12, 13] which is a vital peptide known to play key role in glucose homeostatic as well as other important biological function[14, 15]. Impairment in overall insulin signalling with respect to both insulin secretion and insulin action ultimately affects the blood glucose level resulting in a condition referred to as Diabetes mellitus[16].

Diabetes mellitus is the most common and predominant group of endocrinological disorder affecting people worldwide and thus has been a subject of extensive research for development of number of anti-diabetic treatments. The main aim of anti-diabetic therapy is chiefly to enhance insulin signalling, either by direct injection of insulin, by triggering the downstream targets of the insulin receptor (IR) signalling cascade, or by activating the production or secretion of endogenous insulin[16–18]. One such approach to enhance insulin signalling could be inhibition of Insulin degrading enzyme (IDE) owing to its significant role in insulin catabolism [19]. An early *in vivo* study marked significantly the role of Inhibitor of IDE to potentiate the hypoglycemic action of insulin[20]. Thus following the discovery of IDE in 1949, inhibition of IDE-mediated insulin catabolism has attended considerable attention towards the development of pharmacological inhibitors of IDE to be used as an anti-diabetic therapy[21, 22].

In this *insilico* work, we have modeled the LT10 peptide structure, followed by identification of IDE as one of its novel potential target and further developed suitable peptidomemtics of LT10 peptide. Molecular docking and MD simulation studies were carried out to study the interaction of IDE—LT10 complex which gave an insight into vital interactions. These interaction studies not only revealed the relatively stable interaction of LT10 peptide with IDE but also highlighted the significance of these interactions in inhibition of IDE. Therefore, suggesting the possible novel role of LT10 peptide as an IDE inhibitor and thus its possible anti-diabetic activity apart from its known anti-lethal activity. Moreover our prediction provides a tremendous scope for experimental validation in future. Furthermore, structure based peptidomimetic studies of LT10 peptide has led to identification of a few peptidomimetics that could successfully dock and showed similar inhibitory interactions with IDE. Thus these peptidomimetics could possibly add to a new class of IDE inhibitor derived from LT10 peptide by further experimental validations. Such validation would certainly add to the therapeutic value of LT10 peptide and aid its clinical relevance.

## Materials and Methods

### Peptide modeling and Target screening

Molecular modeling of LT10 peptide was carried out using PEP-FOLD server (http://bioserv.rpbs.univ-paris-diderot.fr/PEP-FOLD/), an online resource for de novo modeling of 3D conformations for peptides between 9 and 25 amino acids. It uses a hidden markov model-derived structural alphabet of 27 motifs composed of 4 residues. It first determines structural alphabet (SA) letters of the sequence and then builds model by assembling the fragments using a greedy algorithm driven by a coarse-grained force field OPEP (Optimized Potential for Efficient structure Prediction). Starting from an amino acid sequence, PEP-FOLD performs series of 200 simulations and returns the most representative conformations identified in terms of energy and population[23, 24]. It generates clusters of models ranked on the basis of their OPEP (Optimized Potential for Efficient structure Prediction) energy. Top ranked LT10 peptide model having lowest sOPEP score (minimum energy) representing the most stable predicted structure was considered to be the best 3D model generated and selected for further studies. The stability of this best predicted structure was further evaluated by subjecting it to Molecular Dynamic Simulation using Desmond (Maestro-Desmond Interoperability Tools, version 3.1, Schrödinger, New York, NY, 2012)[25]

The selected LT10 peptide model was subjected to ReverseScreen3D server (http://www.modelling.leeds.ac.uk/ReverseScreen3D/index.html) for prediction of its probable binding partners. ReverseScreen3D is a ligand-based reverse virtual screening tool that searches against a biologically-relevant and automatically-updated subset of ligands extracted from the RCSB Protein Data Bank [26] in order to identify potential target proteins that are likely to bind a given compound [27]. It generates up to 25 conformers of the query compound followed by their 2D similarity search against all ligands in the database wherein a single ligand with maximum 2D similarity is selected from each unique target protein binding site in the database. This 2D similarity search is followed by 3D structure-based ligand matching carried out between query compound and each of the previously selected database ligands. Based on this 3D alignment score, a ranked list of potential targets thus screened is generated as the output wherein the top ranked target represent highest 3D score. IDE (PDB ID: 3E4A) was amongst the top 15 ranked targets obtained in screening and in the view of its crucial role in Insulin regulation and thereby diabetes [19], it was selected for studying its interaction with LT10 peptide.

### Molecular Docking

Selected LT-10 peptide model was processed in Protein Preparation Wizard 2.2 (Epik Version 2.3, Schrödinger, LLC, New York, 2012). After preparation, LT10 peptide model was subjected to the conformational search of MacroModel (version 9.9,Schrödinger, LLC, New York, NY, 2012) for generating all possible energetically minimum conformers which were then docked on to IDE using Glide dock (Schrödinger, LLC, New York, NY, 2012)[28, 29]. The crystal structure of IDE (PDB ID: 3E4A) obtained from Protein Data Bank (http://www.pdb.org/) [26] was subjected to loop modeling in order to construct its missing C-terminal region from 971 **E** to 978 **I**. This region (^971^ **EFPAQNDI** ^978^) was modeled and the loop was refined using Prime 3.0 (Schrödinger, LLC, NewYork, 2011), followed by MD simulation of 5ns using DESMOND 2012(Maestro-Desmond Interoperability Tools, version 3.1, Schrödinger, New York, NY, 2012)[25]so as to obtain the minimum energy structure of IDE. This IDE structure was thereafter pre-processed and minimized using Protein Preparation Wizard 2.2 (Epik Version 2.3, Schrödinger, LLC, New York, 2012) after addition of H-atoms. Molecular Docking was initiated by generating Grid file (input) using Receptor Grid Generation panel of Glide. Grid file contains receptor (protein structure) and binding site information required for Molecular docking. Extra precision method (XP)[30] of Glide dock[28, 29] was used for docking and sampling of peptide was kept flexible during docking.

Further ahead, set of known IDE inhibitors and MMP (Matrix Metalloprotease) inhibitors [31] were successively docked with IDE (PDB ID: 3E4A) to study the trend in their IC50 values and docking scores.

### Molecular Dynamic (MD) Simulation

Docking results were analyzed for selection of best docked pose of IDE−LT10 complex based on the Glide XP score. Further, this complex was subjected to MD simulation performed using Desmond 2012 (Maestro-Desmond Interoperability Tools, version 3.1, Schrödinger, New York, NY, 2012) [25]. Optimized Potentials for Liquid Simulations (OPLS)[32, 33] all-atom force field was applied to evaluate the stability. The protein structures were solvated with Monte Carlo simulated TIP3P[34] water model in an orthorhombic box with buffer space of 10 Å from the edges of protein. The pressure was controlled using Martina—Tobias—Klein method[35] and the constant simulation temperature was maintained using Nose—Hoover thermostats[36]. Multistep RESPA integrator[37] was used to integrate the equations of motion with an inner time step of 2.0 fs for bonded interactions and non-bonded interactions within the short-range cut-off. For non-bonded interactions beyond the cut-off, an outer time step of 6.0 fs was used. These periodic boundary conditions were applied throughout the system. The default Desmond protocol which includes a series of restrained minimizations and MD Simulations was applied to equilibrate these prepared systems. Two rounds of steepest descent minimization were carried out with a maximum of 2000 steps and a harmonic restraint of 50 kcal/mol/Å^2^ on all solute atoms followed by a series of four MD simulations. The first simulation was 12 ps run, at a temperature of 10 K in the NVT (constant number of particles, volume, and temperature) ensemble with solute heavy atoms restrained with force constant of 50 kcal/mol/Å^2^. Similarly, the second simulation was also a 12 ps run except it was run in the NPT (constant number of particles, pressure, and temperature) ensemble. This was followed by a 24 ps simulation with an increased temperature of 300 K in the NPT ensemble and with the force constant retained. The last one was a 24 ps simulation at 300 K in the NPT ensemble without restraints. This default equilibration was followed by a 5000 ps NPT simulation to equilibrate the system. The equilibrated system was simulated for the period of 30ns using NPT ensemble with trajectory saved in every 5 ps of time intervals.

MD simulation was analyzed using the analytical tools in the Desmond package. In MD simulation quality analysis, potential energy of the protein as well as total energy of the entire system was calculated. Stability of the docked complex across the trajectory was evaluated from their RMSD (root mean square deviation) plots. RMSF plot (root mean square fluctuation) of the backbone atoms of each residue from its time-averaged position was generated. Further, docked complex structures at regular time interval of MD simulation was extracted and subjected for interaction analysis. These interactions were plotted using LIGPLOT[38]. Snap shot of the protein-peptide complexes were generated using PyMOL v 1.3[39].

MD simulation in Desmond with the same default settings as described above was used to evaluate the stability of best predicted structure of LT10 peptide generated by PEP-FOLD. The system was simulated for a period of 25 ns and the RMSD plot generated was analysed.

### Computational alanine scanning

Alanine-scanning mutagenesis is a simple and widely used method for determining the functional contribution of protein residues. LT10 peptide within IDE-LT10 docked complex was subjected to Alanine-scanning mutagenesis using “Calculate Mutation Energy (Binding)” protocol of Discovery Studio 3.0 (Discovery Studio 3.0, Accelrys Inc., San Diego, CA, USA), in order to identify computationally derived interaction hot spots. This protocol evaluates the effect of single-point mutations on the binding affinity of protein complexes by mutating a set of selected amino-acid residues to one or more specified amino-acid types (amino-acid scanning mutagenesis). The difference between the binding free energy of mutated structure and wild type protein provides the energy effect of each mutation on the binding affinity (mutation energy-ΔΔGmut):

ΔΔGmut=ΔΔGbind(mutant)−ΔΔGbind(wildtype)

The binding free energy is calculated using CHARMM force field as the difference between the free energy of the complex and unbound state. The total energy reported is an empirical weighted sum of van der Waals interaction, electrostatic interactions, an entropy contribution related to the changes in side-chain mobility, and a non-polar, surface dependent, contribution to solvation energy.

### Structure based peptidomimetics design

Set of structure based peptidomimetics were designed from LT10 peptide using SuperMimic software[40]. This software identifies and inserts suitable compounds *i*.*e* spacers that mimic a part or specific position in protein. Since these spacers are characterized by mimicking secondary structure of give peptide, they are also referred to as Secondary Structure Mimetics (SSMs). The screening for spacer at a given position in template peptide is based on spatial superposition of four stem atoms of template (N and Cα atom of first residue and Cα and C atom of the last residue) with the analogous atoms of suitable (mimetic) spacer. The screening library comprises of peptidomimetic building blocks or SSMs collected from the literature such as D-amino acids, α-helices, β-strand, β-turn, γ-turn and peptide mimetics extracted from PDB crystal structure complexes.

From amongst the screened spacers/mimetics obtained for specific positions of LT10 peptide, those having least RMSD of their main chain atoms from that of template peptide were further selected. These selected spacers were inserted at respective position in LT10 peptide by replacing corresponding residue followed by capping peptide termini using Discovery Studio 3.0 (Discovery Studio 3.0, Accelrys Inc., San Diego, CA, USA). The N- and C- termini were capped by acetyl and amino groups respectively to maintain neutral charge. Two types of peptidomimetics were designed; Type 1 with single spacer and Type 2 with multiple spacers. All these peptidomimetics were subjected to steepest decent minimization of 200 steps using CharMM force field and Generalized Borne as implicit solvent model.

All these designed peptidomimetics were further prepared in LigPrep (version 2.5, Schrödinger, LLC, New York, NY, 2012) followed by docking to IDE using Glide Extra-Precision (XP) mode[30] with the same receptor grid used earlier.

## Results and Discussion

### Prediction of suitable LT10 Peptide model and its potential protein targets

**Fig 1:**
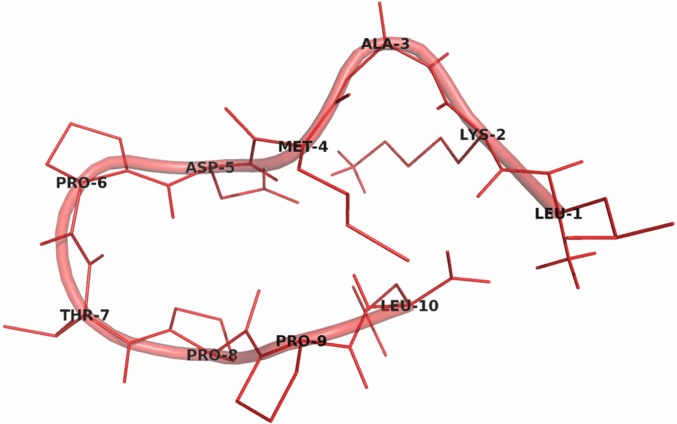
Model of LT10 synthetic peptide as viewed in PyMol.

LT10 synthetic peptide (LKAMDPTPPL) derived from N-terminal of LTNF was modeled (Fig. 1) using PEPFOLD server that predicts the *de novo* peptide structure from given amino acid sequence[24]. It returns an archive of all the generated models, the detail of the clusters and the best conformation from 5 best clusters. These clusters are ranked based on the sOPEP (Optimized Potential for Efficient structure Prediction) energies of their representative models. The model with lowest sOPEP score is considered to be the best model generated. For, LT10 synthetic peptide, 48 clusters of models was generated and model from the 1^st^ cluster with lowest sOPEP score of -5.77 was selected for further screening. The stability of this predicted structure was analysed by subjecting it to MD simulation of 25ns using Desmond [25]. The RMSD plot obtained along the trajectory of simulation (S1 Fig.) showed relatively stable region 17ns onwards. Further, the comparative structural analysis (in PyMol) of peptide conformations extracted along the stable region of trajectory with the best predicted model from PEP-FOLD showed RMSD of ~0.5 Å. Thus this evaluation indicates the possible relative structural stability of predicted LT10 peptide model.

The LT10 peptide model was then screened for identification of its potential targets using ReverseScreen3D server [27]. The potential targets obtained in screening are ranked based on the 3D alignment score of ligand (in complex with target) with the query compound/ligand. Thus the top ranked target represents the one with highest 3D score. IDE [PDB ID: 3E4A] was among the top 15 ranked targets obtained in screening (S1 Table). IDE was selected for our studies owing to its crucial role in insulin regulation and thereby diabetes [19, 20]. Moreover, the earlier studies investigating the role of LT10 peptide for its possible therapeutic role in diabetes although with different target [6–8]was also a major factor that led to investigating the novel interaction of LT10 peptide with IDE.

### Molecular docking and MD simulation analysis: Dictating LT10 peptide’s relative stability of interaction

**Fig 2:**
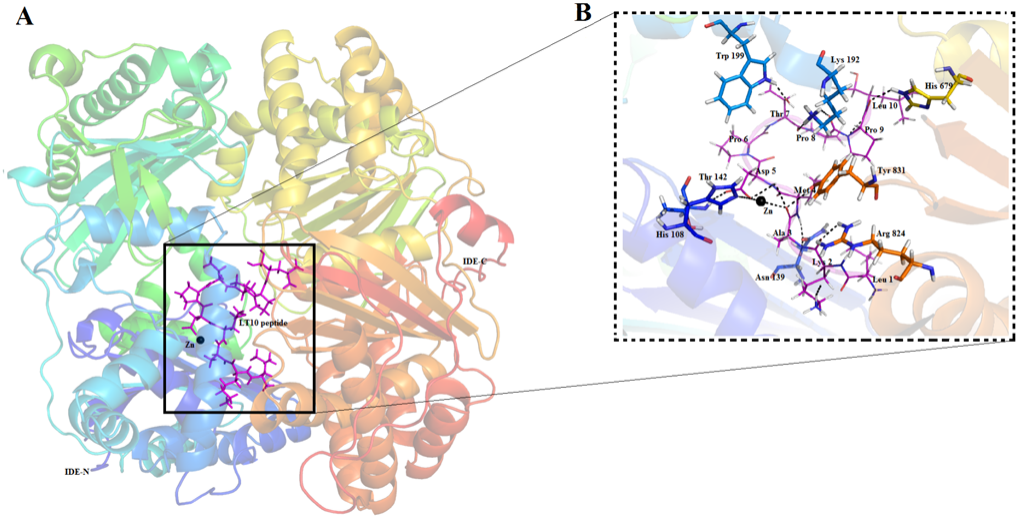
IDE-LT10 docked complex. **(A)** Overall structure of LT10 peptide bound to IDE. IDE represented as cartoon with labelled N- and C- termini with catalytic zinc as black sphere and LT10 peptide represented as (magenta) sticks **(B)** Interaction details showing LT10 peptide as labelled (magenta) lines interacting with IDE shown as labelled (Green) stick.

Extra Precision (XP) method is highly accurate and generated poses during docking that were ranked based on the XP Glide score. Docked pose of IDE-LT10 complex with lowest XP Glide score was considered to be the best pose (Fig. 2) and further refined by MD simulation. LT10 peptide was docked to IDE with a highly significant Glide score of -14.697 Kcal/mol. These interactions along with the Glide score and Emodel values are given in Table 1. This molecular interaction analysis showed LT10 peptide to be involved in hydrogen bonding with His 108, Asn 139, Thr 142, Lys 192, Trp 199,His 679,Arg 824,Tyr 831, Zn 2000 residues and hydrophobic interactions with His 112,Phe 115, Phe 141, Gln 680, Phe 820, Ile 832 residues of IDE (S2 Fig.).

**Table 1:** IDE-LT10 complex: best docked pose analysis.

Docked complex	Interacting residues		Glide score (Kcal/mol)	Emodel value(Kcal/mol)
	H-bond Interactions	Vdw Interactions		
IDE-LT10	His 108(A), Asn 139(A),Thr 142(A), Lys 192(A),Trp 199(A), His 679(A),Arg 824(A), Tyr 831(A),Zn 2000(A),	His 112(A), Phe 115(A),Phe 141(A),Gln 680(A),Phe 820(A), Ile 832(A),	-14.697	-84.591

**Fig 3:**
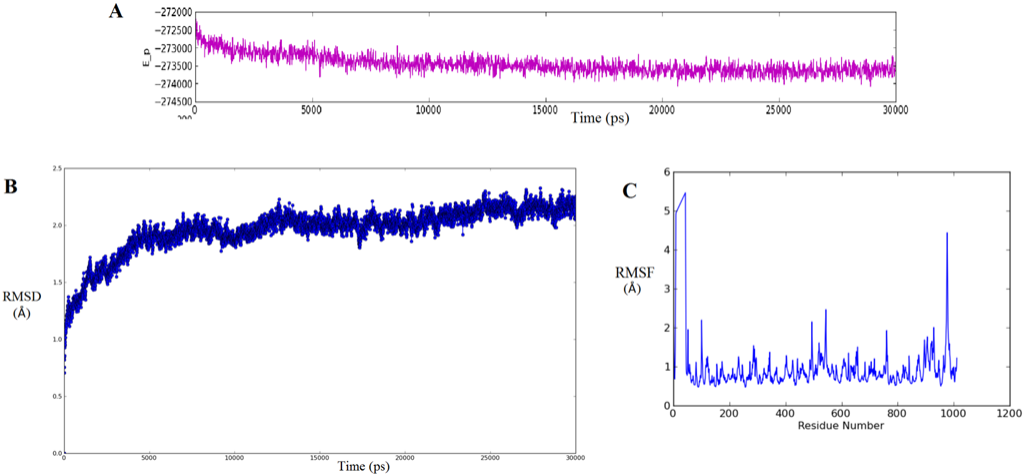
MD simulation (30ns) trajectory analysis of IDE-LT10 complex. **(A)**The potential energy plot of IDE-LT10 complex showing relatively stable complex system.**(B)** RMSD plot representing deviation of less than 1 A° along the stable region preceded by small rearrangement from the initial conformation **(C)** RMSF graph representing the extent of conformational arrangement upon LT10 peptide binding to IDE.

The stability of this docked complex system was monitored via MD simulation of 30ns. The trajectory was analysed for stability through potential energy and RMSD(root mean square deviation) plots and fluctuation via RMSF (root mean square fluctuation) graph. The potential energy plot (Fig. 3A) indicated well equilibrated and relatively stable complex system, throughout the simulations. RMSD during simulation was calculated with respect to their initial docking structure. It provided quantitative output of deviations with respect to time. The RMSD plot (Fig. 3B) showed a relatively stable region preceded by rapid increase during the first few hundred picoseconds. The initial small rearrangement of conformation explains the rapid increase, which is followed by a continuous stretch of stable trajectory with minimum deviation observed. This deviation of less than 1 Å suggests fairly stable binding of LT10 peptide to IDE. Further, root-mean-square fluctuation (RMSF) of the backbone atoms of each residue from its time-averaged position was examined via RMSF graph (Fig. 3C). It showed higher relative fluctuations around the N- and C-termini of IDE, followed by small peaks in between. These fluctuations mark the extent of conformational arrangement upon LT10 peptide binding. The RMSF graph thus suggests that LT10 binding to IDE has relatively minor influence on the internal region but relative effect on external loops that are located far away from the substrate binding site.

**Fig 4:**
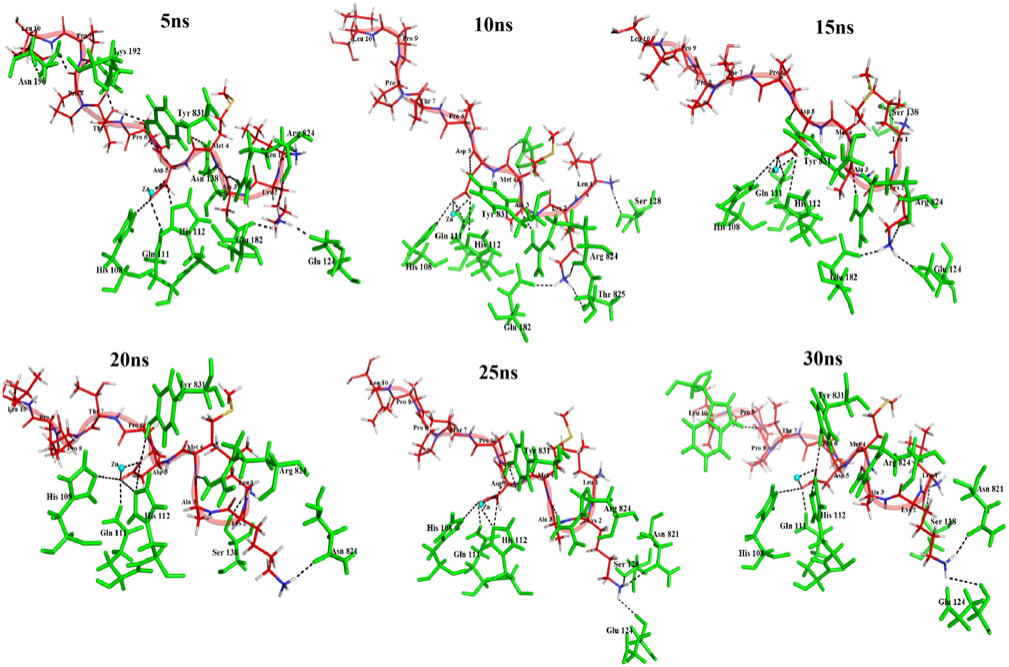
Interaction analysis of IDE-LT10 complex at regular intervals of 30ns MD simulation. IDE-LT10interaction at 5ns, 10ns, 15ns, 20ns, 25ns, and 30ns. LT10 peptide represented as red stick and interacting residues of IDE as green sticks. Catalytic zinc is represented as cyan sphere.

Interactions at different time point of MD simulation (5ns, 10ns, 15ns, 20ns, 25ns, and 30ns) were computed and analysed (Fig. 4). This analysis revealed that residues His 108, Gln 111,His 112, Glu 124, Arg 824,Tyr 831, and Zn 2000 of IDE are involved in hydrogen bond (H-bond) interaction with LT10 peptide throughout the simulation. The residue Asn 821 appears to show H-bond interactions across the stable trajectory since 15ns of simulation. IDE residues Phe 202, Phe 820 and Ile 832 appeared consistently in hydrophobic interactions with LT10 peptide along the stable region of trajectory (S3 Fig.). Further, the behaviour of LT10 peptide across the simulation trajectory at these regular intervals was analysed by studying its molecular interaction and structural deviation. LT10 residues namely Leu1, Lys 2, Ala 3, Asp 5 are consistently involved in hydrogen interaction, whereas others showed few hydrophobic interactions (S3 Fig.). The RMSD of LT10 peptide at these regular time intervals of MD simulation with respect to its conformation in docked structures was calculated (using PyMOL). It showed RMSD of 4.1 Å, 4.5 Å, 4.7 Å, 4.7 Å, 4.6 Å and 4.8 Å at 5ns, 10ns, 15ns, 20ns, 25ns and 30ns respectively. Despite of this significant deviation from docked complex, the LT10 peptide shows relatively less deviation along with consistent interaction across the MD simulation trajectory. Thus the overall MD Simulation trajectory analysis along with comparative interaction analysis at its regular time intervals indicates relatively stable binding of LT10 peptide to IDE.

### Molecular Basis of IDE inhibition by LT10 peptide: Insights from comparative interaction studies

Insulin Degrading Enzyme—IDE (EC 3.4.24.56) is a prime regulator of insulin degradation and inactivation[11, 12, 41]. IDE is 110-kDa metallopeptidase that belongs to small superfamily M16 of Zinc metalloproteases, referred to as “inverzincins” since they are characterized by presence of a zinc-binding motif (HxxEH) that is inverted with respect to that within conventional zinc-metalloproteases (HExxH) [42, 43]. The crystal structure of IDE (monomer) consists of two bowl shaped halves referred to as N- and C- terminal halves (IDE-N and IDE-C) together composed of four domains (Fig. 2A). Domain 1 (residues 43–285) and Domain 2(residues 286–515) make up the N terminal region whereas domain 3 (residues 542–768) & 4 (residues 769–1016) make up the C-terminal region. These N-and C-terminal halves are connected by a flexible linker which allows IDE to adopt an ‘‘open” and “closed” conformation. Open-conformation permits the entry of substrates and exit of products whereas the closed-conformation entraps the substrates completely within an unusually large internal chamber. This enclosed chamber, also referred to as catalytic chamber encompasses the catalytic site. The catalytic site contains Zinc ion coordinated by zinc-binding motif (HxxEH) that comprises of two histidine (His108 and His 112) and one glutamate (Glu 111)[11, 41–43].

Inhibitors of IDE have been studied for their possible use as one of the anti-diabetic treatment owing to their potential in enhancing insulin signalling [18, 19]. Recently, peptide hydroxamate IDE inhibitor (referred to as**Ii1**)has been described as the first potent and selective small molecule inhibitor of IDE [31]. The interactions of IDE-Ii1complex structure were compared with the consistent interactions observed across the MD simulation trajectory of IDE-LT10 complex. This comparative interaction analysis (Table 2) revealed many common interactions *viz*. His 108, Gln111,His 112, Arg 824, Tyr 831,Zn 2000 involved in H-bond interaction and Phe 820 involved in hydrophobic interaction. This sharing of interactions, significant for IDE inhibition might direct towards the possible role of LT10 peptide as an IDE-inhibitor. Moreover, LT10 peptide showed better docking score as compared to Ii1 with IDE upon docking (Table 2).

**Table 2:** Comparative interaction analysis of LT10 peptide and known potent IDE-inhibitor (Ii1) docked to IDE, respectively.

IDE complex	Interacting residues		Glide score (Kcal/mol)
	H-bond Interactions	Vdw Interactions	
IDE-LT10	His 108*, Gln111*,His 112*,Glu 124,Asn 821,Arg 824*,Tyr 831*,Zn 2000*	Phe 202,Phe 820*,Ile 832	-14.697
IDE-Ii1	His 108*, Gln 111*, His 112*,Asn 139, Glu 189, Arg 824*,Tyr 831*, ZN 2000*	Phe 115, Leu 116, Ser 128,Ala 140, Glu 182, Phe 820*	-9.683

Note: common interacting residues are represented by *

These comparative interaction studies with the known potent IDE inhibitor (Ii1) as well as interaction analysis at different time point of simulation (Fig. 4) highlight the vital interactions that contribute significantly towards inhibition of IDE. Particularly, Asp5 residue of LT10 peptide shows hydrogen bond interactions with catalytic zinc as well as the zinc binding motif (HxxEH) *viz*. His 108, Gln 111 and His 112 of IDE which may possibly led to inhibition of its enzymatic activity. Another important set of interactions occur at C-terminal region of IDE, wherein LT10 residues mostly Leu 1, Lys 2, Ala 3 shows H-bond interaction with Asn 821,Arg 824, Tyr 831 and hydrophobic interactions with Phe 202, Phe 820, Ile 832(S3 Fig.). This binding to C-terminal region renders IDE in close state. Thus LT10 peptide binds to both catalytic as well as C-terminal region, holding IDE in a closed inactive state thereby possibly making the catalytic chamber unavailable for other substrate of IDE.

### LT10 peptide predicted to bind better than known IDE inhibitors

**Fig 5:**
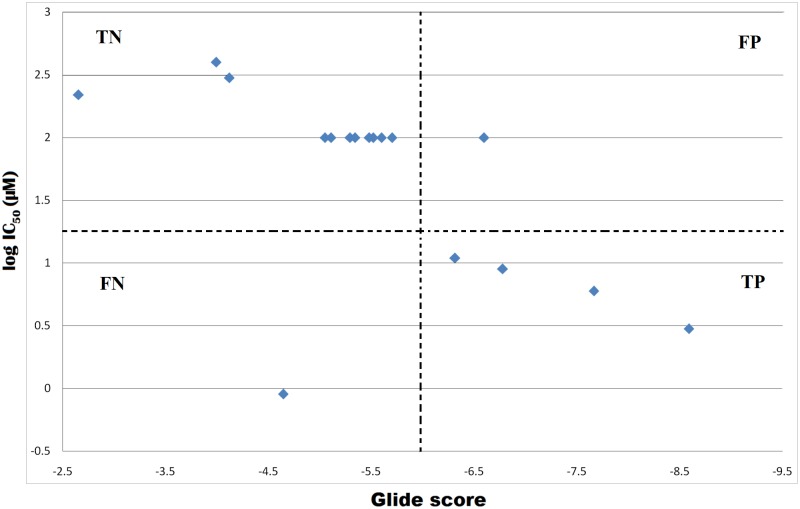
Plot of experimentally determined log IC50 values of 17 compounds versus their Glide docking scores.

Few known IDE inhibitors and a set of MMP inhibitors experimentally studied for their IDE inhibitory activity [31] were docked with IDE in-order to validate the docking protocol. All these molecules are listed (Table 3) along with their IC50 values, log IC50 values, computed Glide docking scores and emodel values. Of these 17 compound studied, 5 had IC50 value <12μM and were classified as active (true positive) and remaining 12 compounds with IC50 value >100 μM were classified as less active/ inactive (true negative). Of the 5 actives compounds, total 4 were correctly predicted as true positives, whereas 1 (nullscript)with least IC50 value0.9μMwas wrongly predicted as false negatives owing to its poor docking score. Similarly, of the 12 less active/inactive compounds, 10 were correctly predicted as true negative, whereas 2 compounds (MMP-2/9 Inhibitor II and N-ethylmaleimide) were wrongly predicted as false positive (Fig. 5). MMP-2/9 Inhibitor II although classified into inactive compounds based on its IC50 value, showed docking score favourable to fit in the active class. Similarly, N-ethylmaleimide with IC50 value of 220 μM showed less efficient docking as compared to others with greater IC50 values. Thus, the accuracy of docking protocol was calculated to be ~0.7.

$$Accuracy\;=\;\frac{Truepositive\;(TP)\;+Truenegative\;(TN)}{Truepositive\left(TP\right)+\;Falsenegative\left(FN\right)+\;Falsepositive\left(FP\right)+\;Truenegative\;(TN)}$$

**Table 3:** List of ligands studied for their IDE inhibitory activity.

IDE inhibitors	IC50 (μM)	Log IC50	Glide score (Kcal/mol)	Emodel value(Kcal/mol)
Nullscript	0.9	-0.04	-4.645	-67.213
TAPI-1	3	0.47	-8.588	-99.271
GM6001 (Galardin)	6	0.77	-7.665	-93.611
TAPI-0	9	0.95	-6.776	-81.261
TAPI-2	11	1.04	-6.313	-72.261
MMP-9 Inhibitor I	>100	2	-5.518	-83.6791
MMP-3 Inhibitor VII	>100	2	-5.478	-69.413
MMP-2/9 Inhibitor II	>100	2	-6.593	-70.429
MMP-9/13 Inhibitor II	>100	2	-5.702	-86.299
MMP-9/13 Inhibitor I	>100	2	-5.599	-76.119
MMP-3 Inhibitor II	>100	2	-5.107	-55.099
MMP-8 Inhibitor I	>100	2	-5.291	-58.899
MMP Inhibitor II	>100	2	-5.340	-82.033
MMP-2/9 Inhibitor IV	>100	2	-5.049	-55.457
N-ethylmaleimide	220	2.34	-2.652	-27.054
1,10-Phenanthroline	300	2.47	-4.123	-34.690
Bacitracin	400	2.60	-3.991	-33.791

For potent IDE inhibitor-Ii1, the IC50 value has not been provided, but has been studied for its potent inhibition of IDE. This can be observed from its docking score (-9,683 kcal/mol) which is more than other compared inhibitors (Table 3). However, LT10 peptide shows the highest docking score of all (-14.679 kcal/mol) and thus it might exhibit better binding affinity for IDE as compared to other IDE inhibitors compared. It assumes importance to consider docking results for binding affinity from a cautionary perspective, especially in the context of observing the score for LT10 peptide and peptidomimetics (discussed further) in comparison to that from the sphere of known IDE inhibitors since Glide probably has been trained on small molecules.

### New set of putative IDE inhibitors derived from *in silico* peptidomimetic studies of LT10 peptide

Alanine is a widely preferred substitution residue in mutagenesis studies owing to its structure that eliminates the side chain beyond β carbon and yet does not alter the main-chain conformation nor does it impose extreme electrostatic or steric effects [44]. The computation based alanine-scanning mutagenesis of LT10 peptide was carried out using “Calculate Mutation Energy” protocol of Discovery studio 3.0 (Discovery Studio 3.0, Accelrys Inc., San Diego, CA, USA). It computed mutation energy for substitution at each position based on which the effect of respective mutation on binding stability with IDE was reported (Table 4). The LT10 residues (^1^ **LK** ^2^,^5^ **DP** ^6^,^9^ **PL** ^10^) with destabilizing effect upon alanine mutation were considered as hot spot residue for IDE interaction and therefore kept unaltered for peptidomimetic design. Moreover, interaction analysis (S3 Fig.) also revealed LT10 residues- Leu 1, Lys 2, Ala 3 and Asp 5 to be consistently involved in hydrogen bonding with IDE which thus confirms theirs significance as key interacting residues. On the other hand, the LT10 residues (^3^ **AM** ^4^, ^7^ **TP** ^8^) with stabilizing effect of computational alanine mutation were used to screen suitable spacers to be replaced with.

**Table 4:** Computational alanine scanning mutagenesis of LT10 peptide.

Mutation	Mutation energy	Effect of mutation
LEU1.ALA	1.66	destabilizing
LYS2.ALA	5.17	destabilizing
ALA3.ALA	0	neutral
MET4.ALA	0.3	neutral
ASP5.ALA	1.73	destabilizing
PRO6.ALA	0.96	destabilizing
THR7.ALA	-0.07	neutral
PRO8.ALA	0.21	neutral
PRO9.ALA	1.08	destabilizing
LEU10.ALA	2.46	destabilizing

**Fig 6:**
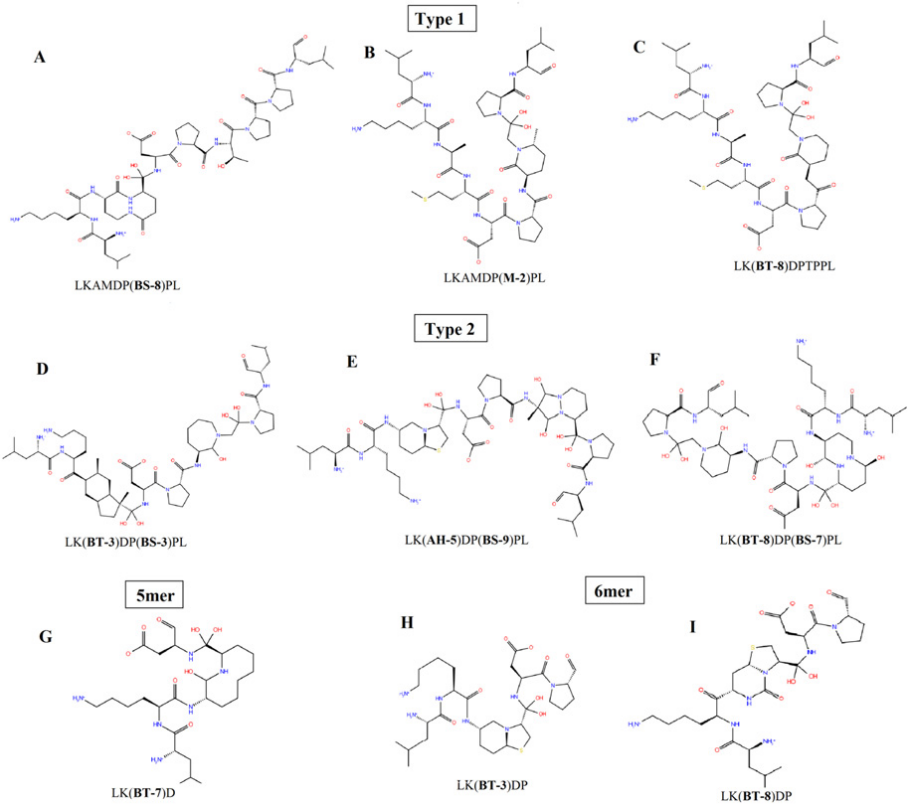
Best peptidomimetics inhibitor of IDE designed from LT10 peptide. The best peptidomimetics are represented in 2D structure along with their labelled sequence including spacer (bold) inserted at desired position. (**A)**, (**B)** and (**C)**Type 1 peptidomimetic with single spacer. (**D)**, **(E)** and (**F)**Type 2 peptidomimetic with multiple spacers. **(G)** 5mer peptidomimetic-Subset of Type 1. **(H)**and**(I)** 6mer peptidomimetic-Subset of Type 1.

**Fig 7:**
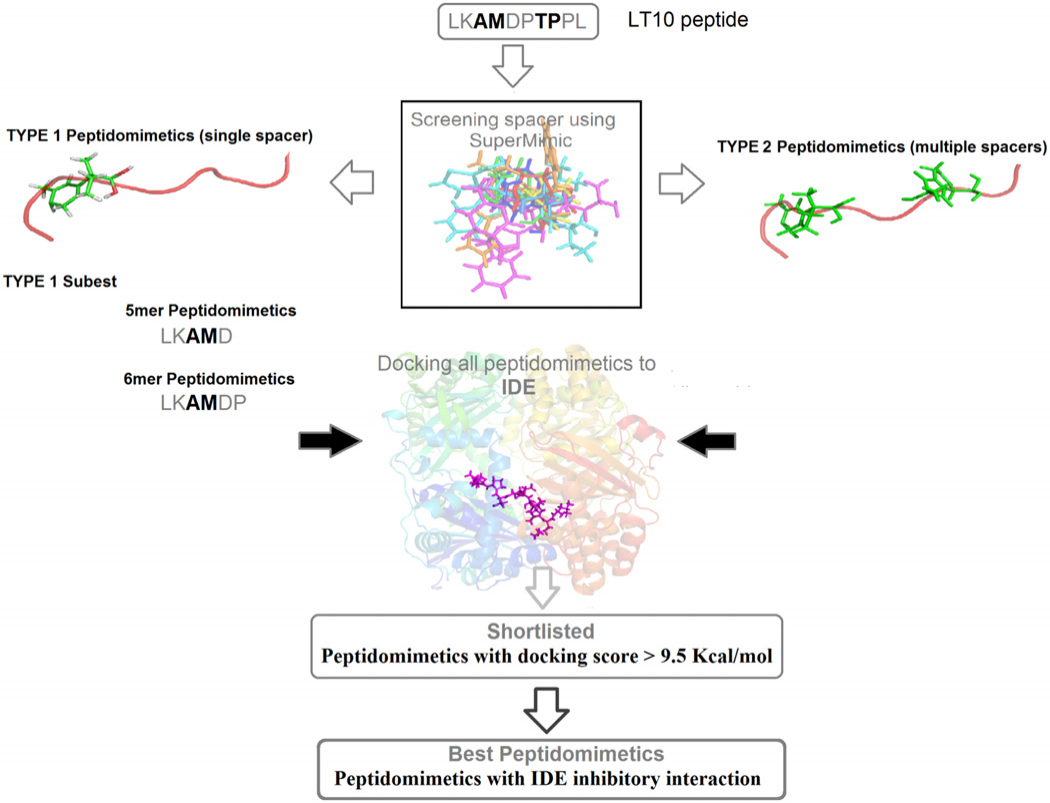
Work flow of peptidomimetic design and selection.

Peptidomimetics are basically the compounds with essential elements (pharmacophore) that mimic a natural peptide or protein in 3D space and retain the ability to interact with the biological target and produce the same biological effect[45]. Suitable spacers were screened from library of Secondary Structure Mimetic (SSMs) using SuperMimic software [40]. Thus series of LT10 peptidomimetics (PM) were designed by inserting these spacers at non hot spot residue positions *i*.*e*.3–4 and 7–8 of LT10 peptide by replacing the respective residues. The designed LT10 peptidomimetics were categorized into two types—Type 1 with single spacer (S2 Table) and Type 2 (S3 Table) with multiple spacers. In addition, two small subsets of peptidomimetics were derived from Type 1 by considering only first 5 and first 6 residues of LT10 peptide *viz*. 5mer and 6mer peptidomimetics with spacer at 3–4 position (S4 Table). All these designed peptidomimetics were docked to IDE using Glide Extra-Precision (XP) mode[30]. From amongst the docked complexes only those with Glide score <-9.5 Kcal/mol were further considered for interaction analysis. The Glide score cut-off was set to <-9.5 since the docking score of known potent IDE inhibitor (Ii1) was calculated to be ~ 9.6 Kcal/mol (Table 2). 11 docked poses from Type 1 and 38 docked poses from Type 2 were shortlisted based on the Glide score cut-off set (Table 5). Similarly 4 docked poses from set of 5mer and 6 docked poses from set of 6mer were shortlisted (Table 6). These short listed poses were further analysed for significant IDE inhibitory interaction using ligplot[37]. Finally, 3 peptidomimetics belonging to Type 1, 3 from Type 2, 1 from 5mer set and 2 from 6mer set were filtered out to be the best peptidomimetics (Fig. 6). The workflow of peptidomimetics design and selection is represented in Fig. 7. The details of these 9 best peptidomimetics along with their Glide score, Glide Emodel value and IDE inhibitory interaction are listed in Table 7. The SMILES notation along with their IUPAC names for each of the best peptidomimetics obtained is provided in S5 Table. The interaction analysis of these best peptidomimetics (S4 Fig.) showed interaction with catalytic Zinc and Zinc binding motif (HxxEH) *viz*. His 108, Gln 111 and His 112thereby,suggesting possible inhibition of IDE. Further they also showed interactions with C-terminal residues of IDE, mainly Phe 820, Arg 824, Tyr 831, Ile 832 thereby probably locking IDE in a closed state. Thus in our analysis, in addition to LT10 peptide, these set of peptidomimetics were found to replicate similar IDE inhibitory interactions as that of known potent IDE inhibitor- Ii1[31].

**Table 5:** Shortlisted, type 1 and type 2 peptidomimetics with their docking details.

Sr.no.	Peptidomimetics	LT10 peptide residue stem atoms	mimetic	RMSD (Å)	Glide Score (Kcal/mol)	Emodel (Kcal/mol)
**Type 1**						
1	BS8	^7^TP^8^	BS-8	0.128	-14.719	-127.715
2	M2	^7^TP^8^	M-2	0.086	-14.328	-130.185
3	M1	^7^TP^8^	M-1	0.06	-14.125	-126.723
4	BT3	^3^AM^4^	BT-3	0.213	-14.115	-148.211
5	BS7	^7^TP^8^	BS-7	0.277	-13.736	-117.152
6	BS13	^3^AM^4^	BS-13	0.062	-13.139	-123.292
7	BS4	^7^T P^8^	BS-4	0.115	-11.742	-142.307
8	AH5	^3^AM^4^	AH-5	0.166	-11.730	-95.347
9	BT7	^3^AM^4^	BT-7	0.164	-11.353	-75.974
10	BT8	^3^AM^4^	BT-8	0.13	-10.435	-101.510
11	BS9	^7^T P^8^	BS-9	0.17	-10.281	-123.236
**Type 2**						
12	MS12	^3^AM^4^	BT-3	0.213	-14.963	-124.157
		^7^TP^8^	BS-3	0.073		
13	MS60	^3^AM^4^	BS-13	0.062	-13.691	-128.778
		^7^TP^8^	BS-9	0.17		
14	MS25	^3^AM^4^	BT-7	0.164	-13.557	-120.863
		^7^TP^8^	BS-7	0.277		
15	MS65	^3^AM^4^	BS-13	0.062	-13.389	-139.956
		^7^TP^8^	M-1	0.06		
16	MS11	^3^AM^4^	AH-5	0.166	-13.306	-101.270
		^7^TP^8^	M-2	0.086		
17	MS56	^3^AM^4^	BS-13	0.062	-13.261	-119.670
		^7^TP^8^	BS-3	0.073		
18	MS34	^3^AM^4^	BT-8	0.13	-13.132	-158.795
		^7^TP^8^	BS-3	0.073		
19	MS1	^3^AM^4^	AH-5	0.166	-13.018	-87.918
		^7^TP^8^	BS-3	0.073		
20	MS15	^3^AM^4^	BT-3	0.213	-12.990	-109.980
		^7^TP^8^	BS-8	0.128		
21	MS66	^3^AM^4^	BS-13	0.062	-12.960	-107.150
		^7^TP^8^	M-2	0.086		
22	MS23	^3^AM^4^	BT-7	0.164	-12.595	-103.903
		^7^TP^8^	BS-3	0.073		
23	MS5	^3^AM^4^	AH-5	0.166	-12.484	-104.301
		^7^TP^8^	BS-9	0.17		
24	MS36	^3^AM^4^	BT-8	0.13	-12.442	-126.948
		^7^TP^8^	BS-7	0.277		
25	MS58	^3^AM^4^	BS-13	0.062	-12.4285	-128.219
		^7^TP^8^	BS-7	0.277		
26	MS27	^3^AM^4^	BT-7	0.164	-12.384	-83.351
		^7^TP^8^	BS-9	0.17		
27	MS32	^3^AM^4^	BT-7	0.164	-12.335	-160.778
		^7^TP^8^	M-1	0.06		
28	MS38	^3^AM^4^	BT-8	0.13	-12.313	-137.014
		^7^TP^8^	BS-9	0.17		
29	MS61	^3^AM^4^	BS-13	0.062	-12.233	-102.370
		^7^TP^8^	PdPP	0.379		
30	MS37	^3^AM^4^	BT-8	0.13	-11.987	-117.550
		^7^TP^8^	BS-8	0.128		
31	MS26	^3^AM^4^	BT-7	0.164	-11.973	-115.387
		^7^TP^8^	BS-8	0.128		
32	MS14	^3^AM^4^	BT-3	0.213	-11.557	-126.786
		^7^TP^8^	BS-7	0.277		
33	MS13	^3^AM^4^	BT-3	0.213	-11.553	-105.148
		^7^TP^8^	BS-4	0.115		
34	MS59	^3^AM^4^	BS-13	0.062	-11.547	-145.841
		^7^TP^8^	BS-8	0.128		
35	MS2	^3^AM^4^	AH-5	0.166	-11.507	-82.886
		^7^TP^8^	BS-4	0.115		
36	MS57	^3^AM^4^	BS-13	0.062	-11.416	-107.253
		^7^TP^8^	BS-4	0.115		
37	MS24	^3^AM^4^	BT-7	0.164	-11.327	-112.881
		^7^TP^8^	BS-4	0.115		
38	MS64	^3^AM^4^	BS-13	0.062	-11.261	10000
		^7^TP^8^	3AIG_I	0.117		
39	MS35	^3^AM^4^	BT-8	0.13	-11.177	-72.906
		^7^TP^8^	BS-4	0.115		
40	MS22	^3^AM^4^	BT-3	0.213	-10.977	-104.007
		^7^TP^8^	M-2	0.086		
41	MS3	^3^AM^4^	AH-5	0.166	-10.934	-66.390
		^7^TP^8^	BS-7	0.277		
42	MS10	^3^AM^4^	AH-5	0.166	-10.871	-94.157
		^7^TP^8^	M-1	0.06		
43	MS6	^3^AM^4^	AH-5	0.166	-10.790	-72.204
		^7^TP^8^	PdPP	0.379		
44	MS28	^3^AM^4^	BT-7	0.164	-10.661	-95.214
		^7^TP^8^	PdPP	0.379		
45	MS4	^3^AM^4^	AH-5	0.166	-10.416	-105.893
		^7^TP^8^	BS-8	0.128		
46	MS17	^3^AM^4^	BT-3	0.213	-10.409	-108.516
		^7^TP^8^	PdPP	0.379		
47	MS33	^3^AM^4^	BT-7	0.164	-10.132	-118.127
		^7^TP^8^	M-2	0.086		
48	MS16	^3^AM^4^	BT-3	0.213	-9.938	-97.006
		^7^TP^8^	BS-9	0.17		
49	MS21	^3^AM^4^	BT-3	0.213	-9.720	-111.739
		^7^TP^8^	M-1	0.06		

**Table 6:** Shortlisted, 5mer and 6mer subsets of type 1peptidomimetics along with their docking details.

No.	Peptidomimetics		LT10 peptide residue stem atoms	mimetic	RMSD (Å)	Glide Score (Kcal/mol)	Emodel (Kcal/mol)
**Type1 subset-5mer**						
1	BT7	^3^AM^4^	BT-7	0.164	-13.526	-97.249
2	BT3	^3^AM^4^	BT-3	0.213	-12.323	-84.312
3	BS13	^3^AM^4^	BS-13	0.062	-11.852	-90.641
4	AH5	^3^AM^4^	AH-5	0.166	-9.801	-81.865
**Type 2 subset-6mer**						
5	BT-3	^3^AM^4^	BT-3	0.213	-12.810	-113.443
6	AH-5	^3^AM^4^	AH-5	0.166	-10.787	-83.998
7	BT-7	^3^AM^4^	BT-7	0.164	-10.719	-113.052
8	1A61_R	^3^AM^4^	1A61_R	0.115	-10.561	-84.447
9	BS-13	^3^AM^4^	BS-13	0.062	-10.413	-126.124
10	BT-8	^3^AM^4^	BT-8	0.13	-10.017	-101.670

**Table 7:** List of best peptidomimetic inhibitors of IDE designed from LT10 peptide, along with their docking and interaction details.

Sr. no.	Peptidomimetics	LT10 peptide residue stem atom	Glide Score (Kcal/mol)	Emodel (Kcal/mol)	H-bond interactions	Hydrophobic interactions
**Type 1**						
1	LKAMDP(**BS-8**)PL	^7^TP^8^	-14.719	-127.715	His 108, His 112,Asn 139, Thr142, Arg 824, Zn 2000,	Phe 115, Leu 116,Phe 820,Tyr 831,Ile 832,Gln 677
2	LKAMDP(**M-2**)PL	^7^TP^8^	-14.328	-130.185	His 108, His 112, Asn 139, Lys 192,Trp 199, Gln 680, Arg 824, Tyr 831, Zn 2000	Phe 115, Phe 141, Glu817,Gln 677,Phe 820
3	LK(**BT-8**)DPTPPL	^3^AM^4^	-10.435	-101.510	Gln 111, His 112,Asn 139, His 679,Arg 824, Zn 2000	Phe 115,Ala 140,Phe 141, Phe 820, Tyr 831,
**Type 2**						
4	MS12LK(**BT-3**)DP(**BS-3**)PL	^3^AM^4^ and ^7^TP^8^	-14.963	-124.157	Gln 111, His 112,Asn 139, Lys 192, Arg 824, Tyr 831, Zn 2000	Phe 115,Ser 128, Leu 131 Ser 138, Trp 199, Met 683,Phe 820, Ile 832,
5	MS5LK(**AH-5**)DP(**BS-9**)PL	^3^AM^4^ and ^7^TP^8^	-12.484	-104.301	His 108,His 112,Gln 111, Ser 138, Asn 139, Ala 140, Lys 192, Arg 431, Arg 824, Zn 2000	Phe 115,Leu 131,Ser 137, Phe 820,Tyr 831,
6	MS36LK(**BT-8**)DP(**BS-7**)PL	^3^AM^4^ and ^7^TP^8^	-12.442	-126.948	Gln 111, His 112,Ser 138, Asn 139, Lys 192,Tyr 831,, Arg 824, Zn 2000	Phe 115,Trp 199, Phe 834,Phe 820,
**Subset -5mer**						
8	LK(**BT-7**)D	^3^AM^4^	-13.526	-97.249	Gln 111,His 112, Ser 138,Arg 824,Tyr 831, Zn 2000	Phe 115,Ser 128,Ser 132,Glu 817,Phe 820,
**Subset 6mer**						
9	LK(**BT-3**)DP	^3^AM^4^	-12.810	-113.443	His 108, His 112, Ser 138, Asn 139, Asn 193, Arg 824, Tyr 831,Zn 2000	Phe 115,Phe 141, Glu 182, Trp 199,
10	LK(**BT-8**)DP	^3^AM^4^	-10.017	-101.670	Gln 111, His 112, Asn 139, Arg 431,Arg 824,Tyr 831,Zn 2000	Phe 115, Phe 820

## Conclusion

LT10 peptide derived from N-terminal of Lethal Toxin Neutralizing Factor (LTNF) isolated from Opossum serum is known for its anti-venom activity. In this work, with an aim to identify novel functionalities of LT10 peptide, Insulin Degrading Enzyme (IDE) was predicted to be its potential target. Our *in silico* analysis revealed relatively stable binding and inhibitory interaction of LT10 peptide with IDE suggesting that LT10 peptide might serve as a novel IDE inhibitor predicted. Since IDE- inhibitors are known to assist treatment of Diabetes mellitus by enhancing insulin signalling; our analysis suggest that LT10 peptide might exhibit this novel mode of anti-diabetic activity apart from its known anti-lethal activity. Furthermore, LT10 peptide was used as a lead for the design of peptidomimetics inhibitors of IDE which showed similar IDE inhibitory interactions. These few peptidomimetics thus obtained, might serve as a set of novel IDE inhibitors derived from LT10 peptide. Our work thus provides great scope for experimental validations. These experimental studies would certainly help validate the novel therapeutic function of LT10 peptide as an anti-diabetic treatment and aid its clinical relevance.

## Supporting Information

S1 Fig RMSD plot of LT10 peptide along the 25ns MD simulation trajectory. (TIF)

S2 Fig Molecular interaction of IDE-LT10 docked complex as plotted in Ligplot. (TIF)

S3 Fig Ligplot of IDE-LT10 interactions at regular interval of 30ns MD simulation. IDE-LT10interactions at 5ns, 10ns, 15ns, 20ns, 25ns, and 30ns. Two-dimensional schematic representation of hydrophobic and hydrogen bond interactions present in docked complex where residues of peptide are shown in purple (Please refer to ‘key’ for details).
(TIF)

S4 Fig Molecular interactions of best peptidomimetics docked to IDE as plotted in Ligplot. (**A)**, (**B)** and (**C)** Type 1 peptidomimetic with single spacer.**(D)**, **(E)** and (**F)** Type 2 peptidomimetic with multiple spacers. **(G)** 5mer peptidomimetic-Subset of Type 1. **(H)** and **(I)** 6mer peptidomimetic-Subset of Type 1. Two-dimensional schematic representation of Hydrophobic and hydrogen bond interactions present in docked complex where residues of peptide are shown in purple (Please refer to ‘key’ for details).
(TIF)

S1 Table List of top 20 targets of LT10 peptide screened from Reverscreen3D. (DOCX)

S2 Table Type 1 peptidomimetics of LT10—with single spacer. (DOCX)

S3 Table Type 2 peptidomimetics of LT10- with multiple spacers (MS). (DOCX)

S4 Table Subset of Type 1 peptidomimetics—5mer and 6mer. (DOCX)

S5 Table Chemical details of best peptidomimetics inhibitors of IDE designed from LT10 peptide. (DOCX)
