# Supplementary figures and images for: An In Silico Insight into Novel Therapeutic Interaction of LTNF Peptide-LT10 and Design of Structure Based Peptidomimetics for Putative Anti-Diabetic Activity
Source: PLoS One. 2015 Mar 27;10(3):e0121860. doi: 10.1371/journal.pone.0121860 (PMC4376886; doi:10.1371/journal.pone.0121860)

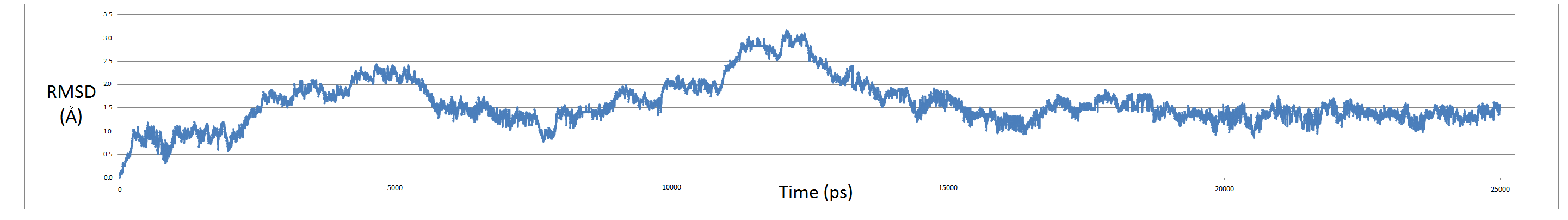

Supplement: S1 Fig — (TIF) [file pone.0121860.s001.tif]

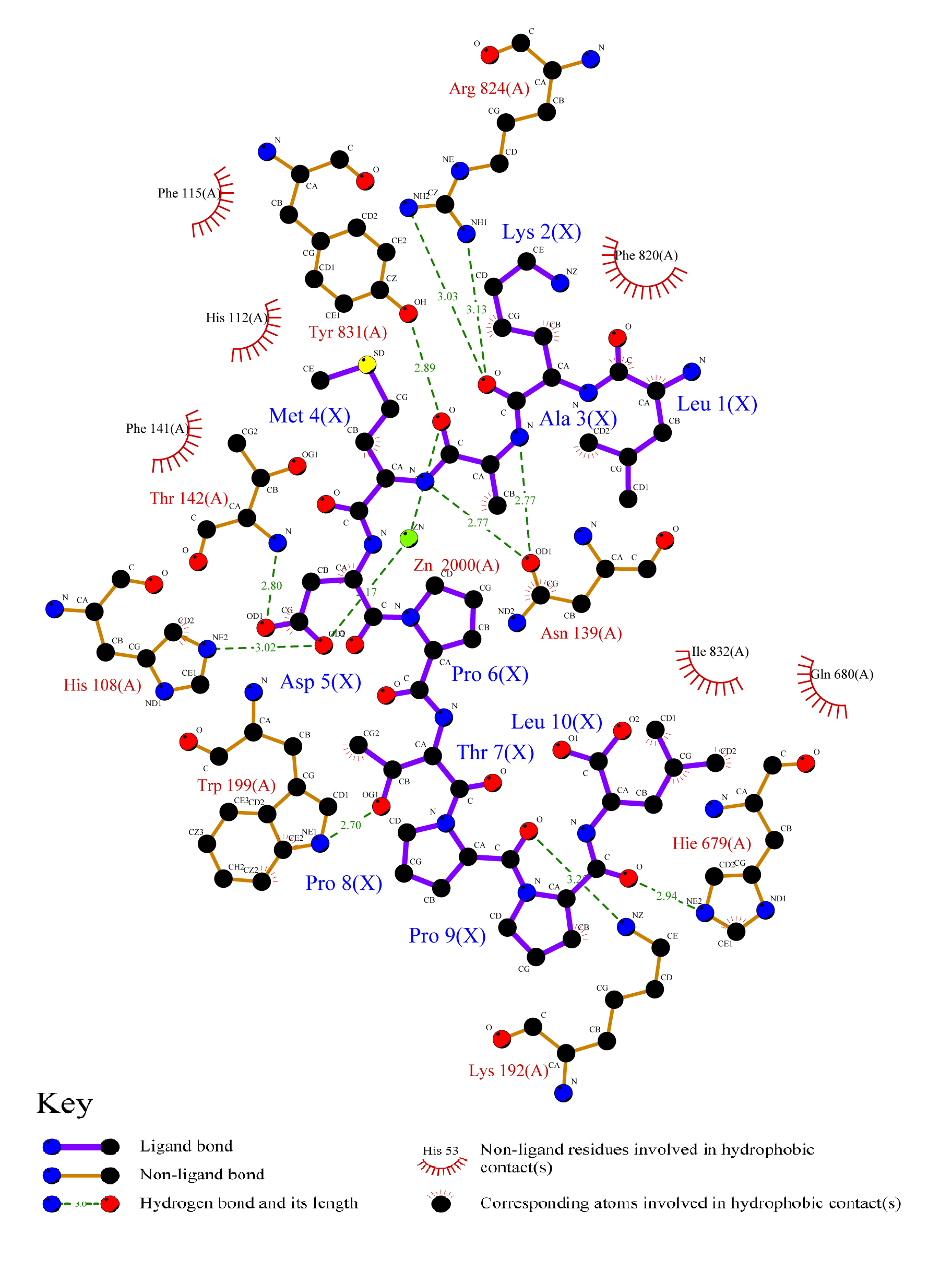

Supplement: S2 Fig — (TIF) [file pone.0121860.s002.tif]

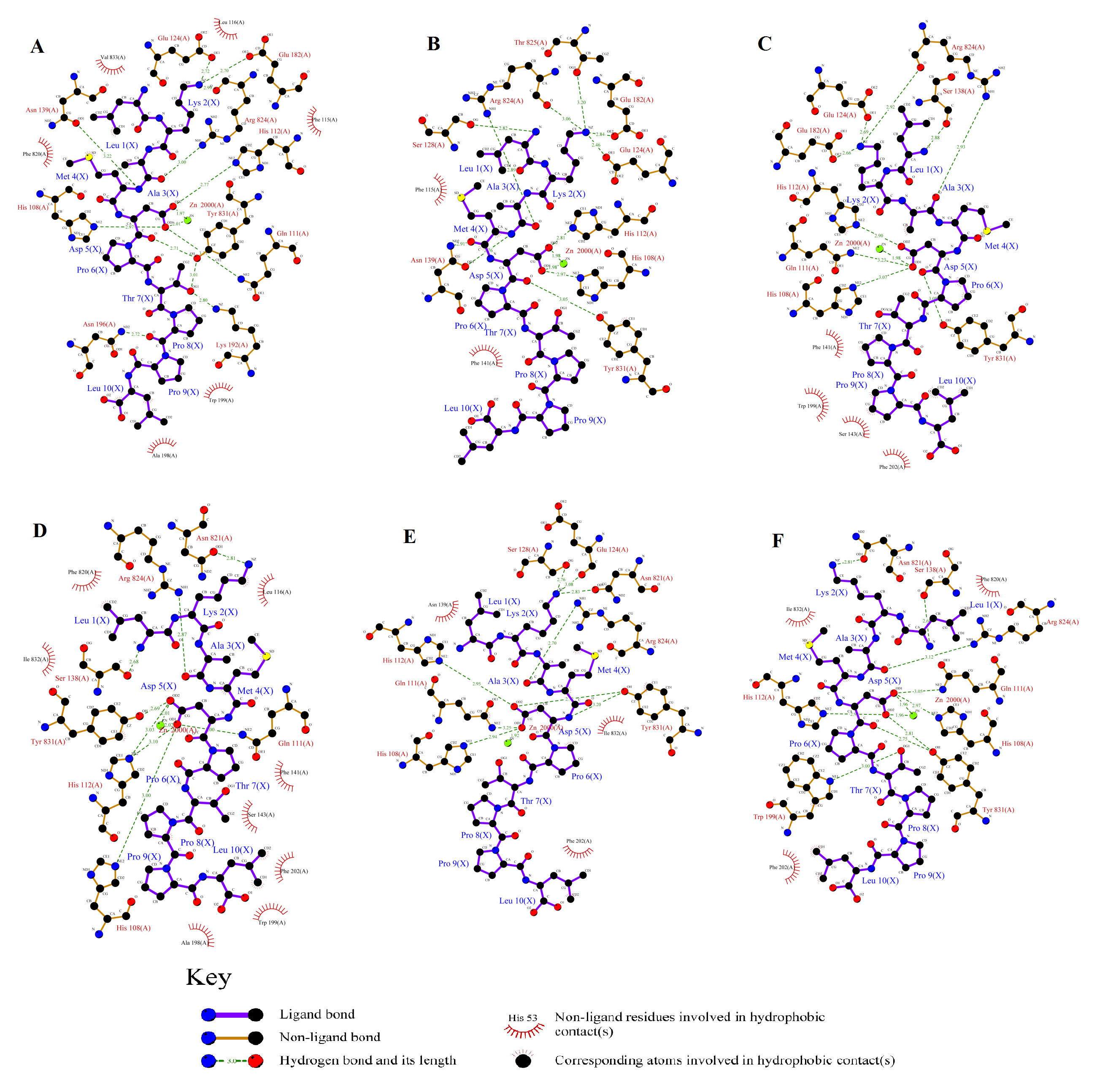

Supplement: S3 Fig — IDE-LT10interactions at 5ns, 10ns, 15ns, 20ns, 25ns, and 30ns. Two-dimensional schematic representation of hydrophobic and hydrogen bond interactions present in docked complex where residues of peptide are shown in purple (Please refer to ‘key’ for details). (TIF) [file pone.0121860.s003.tif]

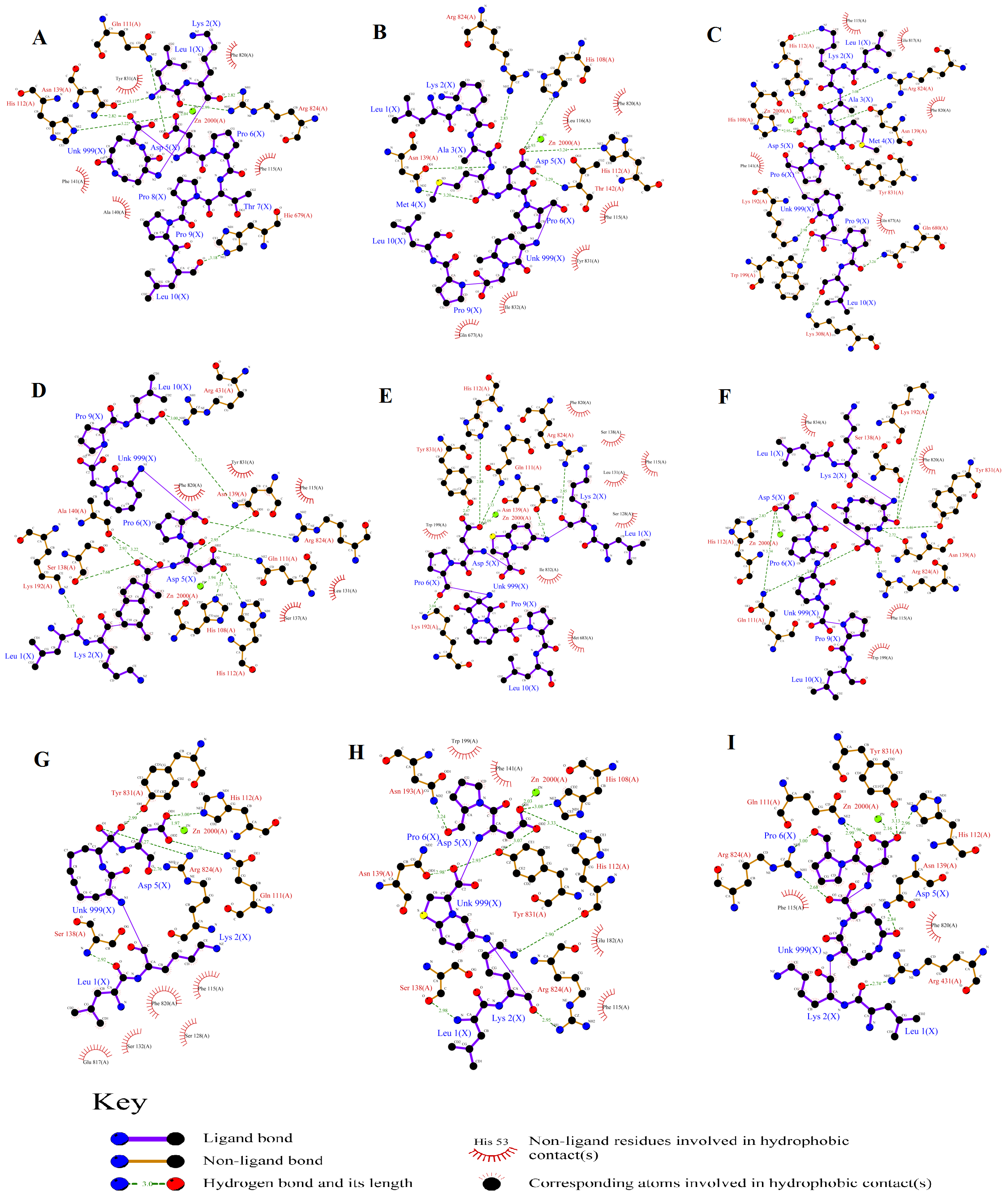

Supplement: S4 Fig — (A), (B) and (C) Type 1 peptidomimetic with single spacer.(D), (E) and (F) Type 2 peptidomimetic with multiple spacers. (G) 5mer peptidomimetic-Subset of Type 1. (H) and (I) 6mer peptidomimetic-Subset of Type 1. Two-dimensional schematic representation of Hydrophobic and hydrogen bond interactions present in docked complex where residues of peptide are shown in purple (Please refer to ‘key’ for details). (TIF) [file pone.0121860.s004.tif]
