# Supplementary material for: An In Silico Insight into Novel Therapeutic Interaction of LTNF Peptide-LT10 and Design of Structure Based Peptidomimetics for Putative Anti-Diabetic Activity
Source: PLoS One. 2015 Mar 27;10(3):e0121860. doi: 10.1371/journal.pone.0121860 (PMC4376886; doi:10.1371/journal.pone.0121860)
Supplement: S1 Table — (DOCX) [file pone.0121860.s005.docx]

**S1 Table. List of top 20 targets of LT10 peptide screened from Reverscreen3D**

| **Rank** | **Cluster name** | **Selected ligand (from PDB entry)** | **2D score** | **3D score** |
| --- | --- | --- | --- | --- |
| 1 | BACULOVIRAL IAP REPEAT-CONTAINING PROTEIN 4 | BI6 (from PDB code 2jk7, chain A) | 0.375 | 0.233333 |
| 2 | SUBTILISIN DY | 1BH (from PDB code 1bh6, chain A) | 0.282723 | 0.224719 |
| 3 | FUCOSE-BINDING LECTIN PA-IIL | 2G0 (from PDB code 3dcq, chain A) | 0.363095 | 0.222222 |
| 4 | BACULOVIRAL IAP REPEAT-CONTAINING PROTEIN 2 | SMK (from PDB code 3mup, chain A) | 0.291005 | 0.217391 |
| 5 | TUBULIN BETA CHAIN | HOS (from PDB code 3du7, chain B) | 0.40081 | 0.214953 |
| 6 | PEPTIDE DEFORMYLASE PDF1 | BB2 (from PDB code 1q1y, chain A) | 0.252525 | 0.214286 |
| 7 | XAA-PRO AMINOPEPTIDASE | ATN (from PDB code 1n51, chain A) | 0.335294 | 0.213483 |
| 8 | BACULOVIRAL IAP REPEAT-CONTAINING PROTEIN 7 | G13 (from PDB code 3f7i, chain A) | 0.317365 | 0.208791 |
| 9 | BACULOVIRAL IAP REPEAT-CONTAINING PROTEIN 4 | X22 (from PDB code 3clx, chain A) | 0.347368 | 0.206522 |
| 10 | PHOSPHOLIPASE A2 | OAP (from PDB code 1kvo, chain A) | 0.289474 | 0.206522 |
| 11 | SUBTILISIN-LIKE PROTEASE | FGK (from PDB code 3i74, chain A) | 0.369318 | 0.202128 |
| 12 | PEPTIDE DEFORMYLASE 1 | BB2 (from PDB code 1ws1, chain A) | 0.406667 | 0.2 |
| 12 | PEPTIDE DEFORMYLASE 1B | BB2 (from PDB code 3m6p, chain A) | 0.406667 | 0.2 |
| 12 | ENDOGLYCOCERAMIDASE II | 16C (from PDB code 2osx, chain A) | 0.253086 | 0.2 |
| 13 | FIBROBLAST COLLAGENASE | RO4 (from PDB code 2tcl, chain A) | 0.365854 | 0.197674 |
| 14 | **INSULIN-DEGRADING ENZYME** | QIX (from PDB code 3e4a, chain A) | 0.291081 | 0.196582 |
| 15 | CASPASE-3 SUBUNIT P12 | 0E4 (from PDB code 3gjs, chain B) | 0.358824 | 0.195652 |
