# Supplementary material for: An In Silico Insight into Novel Therapeutic Interaction of LTNF Peptide-LT10 and Design of Structure Based Peptidomimetics for Putative Anti-Diabetic Activity
Source: PLoS One. 2015 Mar 27;10(3):e0121860. doi: 10.1371/journal.pone.0121860 (PMC4376886; doi:10.1371/journal.pone.0121860)
Supplement: S2 Table — (DOCX) [file pone.0121860.s006.docx]

**S2 Table. Type 1 peptidomimetics of LT10 - with single spacer.**

| **Sr. no.** | **Protein template** | **stem_N** | **stem_C** | **mimetic** | **conformation** | **RMSD(Å)** |
| --- | --- | --- | --- | --- | --- | --- |
| 1 | LT10 peptide | 3 X | 4 X | AH-5 | 1 | 0.166 |
| 2 | LT10 peptide | 3 X | 4 X | BT-3 | 3 | 0.213 |
| 3 | LT10 peptide | 3 X | 4 X | BT-7 | 2 | 0.164 |
| 4 | LT10 peptide | 3 X | 4 X | BT-8 | 9 | 0.13 |
| 5 | LT10 peptide | 3 X | 4 X | BS-12 | 3 | 0.14 |
| 6 | LT10 peptide | 3 X | 4 X | BS-13 | 3 | 0.062 |
| 8 | LT10 peptide | 3 X | 4 X | 1A61_R | 10 | 0.115 |
| 9 | LT10 peptide | 3 X | 4 X | 1W3C_B | 5 | 0.138 |
| 10 | LT10 peptide | 3 X | 4 X | 2AIG_I | 5 | 0.154 |
| 11 | LT10 peptide | 7 X | 8 X | BS-3 | 4 | 0.073 |
| 12 | LT10 peptide | 7 X | 8 X | BS-4 | 1 | 0.115 |
| 13 | LT10 peptide | 7 X | 8 X | BS-7 | 7 | 0.277 |
| 14 | LT10 peptide | 7 X | 8 X | BS-8 | 2 | 0.128 |
| 15 | LT10 peptide | 7 X | 8 X | BS-9 | 8 | 0.17 |
| 16 | LT10 peptide | 7 X | 8 X | PdPP | 1 | 0.379 |
| 17 | LT10 peptide | 7 X | 8 X | 1W3C_B | 3 | 0.165 |
| 18 | LT10 peptide | 7 X | 8 X | 2AIG_I | 10 | 0.118 |
| 19 | LT10 peptide | 7 X | 8 X | 3AIG_I | 6 | 0.117 |
| 20 | LT10 peptide | 7 X | 8 X | M-1 | 4 | 0.06 |
| 21 | LT10 peptide | 7 X | 8 X | M-2 | 3 | 0.086 |
