# Supplementary material for: An In Silico Insight into Novel Therapeutic Interaction of LTNF Peptide-LT10 and Design of Structure Based Peptidomimetics for Putative Anti-Diabetic Activity
Source: PLoS One. 2015 Mar 27;10(3):e0121860. doi: 10.1371/journal.pone.0121860 (PMC4376886; doi:10.1371/journal.pone.0121860)
Supplement: S3 Table — (DOCX) [file pone.0121860.s007.docx]

**S3 Table. Type 2 peptidomimetics of LT10- with multiple spacers (MS).**

| **Peptidomimetics** | **Protein template** | **stem_N** | **stem_C** | **mimetic** | **conformation** | **RMSD(Å)** |
| --- | --- | --- | --- | --- | --- | --- |
| MS1 | LT10 peptide | 3 X | 4 X | AH-5 | 1 | 0.166 |
|  |  | 7 X | 8 X | BS-3 | 4 | 0.073 |
| MS2 | LT10 peptide | 3 X | 4 X | AH-5 | 1 | 0.166 |
|  |  | 7 X | 8 X | BS-4 | 1 | 0.115 |
| MS3 | LT10 peptide | 3 X | 4 X | AH-5 | 1 | 0.166 |
|  |  | 7 X | 8 X | BS-7 | 7 | 0.277 |
| MS4 | LT10 peptide | 3 X | 4 X | AH-5 | 1 | 0.166 |
|  |  | 7 X | 8 X | BS-8 | 2 | 0.128 |
| MS5 | LT10 peptide | 3 X | 4 X | AH-5 | 1 | 0.166 |
|  |  | 7 X | 8 X | BS-9 | 8 | 0.17 |
| MS6 | LT10 peptide | 3 X | 4 X | AH-5 | 1 | 0.166 |
|  |  | 7 X | 8 X | PdPP | 1 | 0.379 |
| MS7 | LT10 peptide | 3 X | 4 X | AH-5 | 1 | 0.166 |
|  |  | 7 X | 8 X | 1W3C_B | 3 | 0.165 |
| MS8 | LT10 peptide | 3 X | 4 X | AH-5 | 1 | 0.166 |
|  |  | 7 X | 8 X | 2AIG_I | 10 | 0.118 |
| MS9 | LT10 peptide | 3 X | 4 X | AH-5 | 1 | 0.166 |
|  |  | 7 X | 8 X | 3AIG_I | 6 | 0.117 |
| MS10 | LT10 peptide | 3 X | 4 X | AH-5 | 1 | 0.166 |
|  |  | 7 X | 8 X | M-1 | 4 | 0.06 |
| MS11 | LT10 peptide | 3 X | 4 X | AH-5 | 1 | 0.166 |
|  |  | 7 X | 8 X | M-2 | 3 | 0.086 |
| MS12 | LT10 peptide | 3 X | 4 X | BT-3 | 3 | 0.213 |
|  |  | 7 X | 8 X | BS-3 | 4 | 0.073 |
| MS13 | LT10 peptide | 3 X | 4 X | BT-3 | 3 | 0.213 |
|  |  | 7 X | 8 X | BS-4 | 1 | 0.115 |
| MS14 | LT10 peptide | 3 X | 4 X | BT-3 | 3 | 0.213 |
|  |  | 7 X | 8 X | BS-7 | 7 | 0.277 |
| MS15 | LT10 peptide | 3 X | 4 X | BT-3 | 3 | 0.213 |
|  |  | 7 X | 8 X | BS-8 | 2 | 0.128 |
| MS16 | LT10 peptide | 3 X | 4 X | BT-3 | 3 | 0.213 |
|  |  | 7 X | 8 X | BS-9 | 8 | 0.17 |
| MS17 | LT10 peptide | 3 X | 4 X | BT-3 | 3 | 0.213 |
|  |  | 7 X | 8 X | PdPP | 1 | 0.379 |
| MS18 | LT10 peptide | 3 X | 4 X | BT-3 | 3 | 0.213 |
|  |  | 7 X | 8 X | 1W3C_B | 3 | 0.165 |
| MS19 | LT10 peptide | 3 X | 4 X | BT-3 | 3 | 0.213 |
|  |  | 7 X | 8 X | 2AIG_I | 10 | 0.118 |
| MS20 | LT10 peptide | 3 X | 4 X | BT-3 | 3 | 0.213 |
|  |  | 7 X | 8 X | 3AIG_I | 6 | 0.117 |
| MS21 | LT10 peptide | 3 X | 4 X | BT-3 | 3 | 0.213 |
|  |  | 7 X | 8 X | M-1 | 4 | 0.06 |
| MS22 | LT10 peptide | 3 X | 4 X | BT-3 | 3 | 0.213 |
|  |  | 7 X | 8 X | M-2 | 3 | 0.086 |
| MS23 | LT10 peptide | 3 X | 4 X | BT-7 | 2 | 0.164 |
|  |  | 7 X | 8 X | BS-3 | 4 | 0.073 |
| MS24 | LT10 peptide | 3 X | 4 X | BT-7 | 2 | 0.164 |
|  |  | 7 X | 8 X | BS-4 | 1 | 0.115 |
| MS25 | LT10 peptide | 3 X | 4 X | BT-7 | 2 | 0.164 |
|  |  | 7 X | 8 X | BS-7 | 7 | 0.277 |
| MS26 | LT10 peptide | 3 X | 4 X | BT-7 | 2 | 0.164 |
|  |  | 7 X | 8 X | BS-8 | 2 | 0.128 |
| MS27 | LT10 peptide | 3 X | 4 X | BT-7 | 2 | 0.164 |
|  |  | 7 X | 8 X | BS-9 | 8 | 0.17 |
| MS28 | LT10 peptide | 3 X | 4 X | BT-7 | 2 | 0.164 |
|  |  | 7 X | 8 X | PdPP | 1 | 0.379 |
| MS29 | LT10 peptide | 3 X | 4 X | BT-7 | 2 | 0.164 |
|  |  | 7 X | 8 X | 1W3C_B | 3 | 0.165 |
| MS30 | LT10 peptide | 3 X | 4 X | BT-7 | 2 | 0.164 |
|  |  | 7 X | 8 X | 2AIG_I | 10 | 0.118 |
| MS31 | LT10 peptide | 3 X | 4 X | BT-7 | 2 | 0.164 |
|  |  | 7 X | 8 X | 3AIG_I | 6 | 0.117 |
| MS32 | LT10 peptide | 3 X | 4 X | BT-7 | 2 | 0.164 |
|  |  | 7 X | 8 X | M-1 | 4 | 0.06 |
| MS33 | LT10 peptide | 3 X | 4 X | BT-7 | 2 | 0.164 |
|  |  | 7 X | 8 X | M-2 | 3 | 0.086 |
| MS34 | LT10 peptide | 3 X | 4 X | BT-8 | 9 | 0.13 |
|  |  | 7 X | 8 X | BS-3 | 4 | 0.073 |
| MS35 | LT10 peptide | 3 X | 4 X | BT-8 | 9 | 0.13 |
|  |  | 7 X | 8 X | BS-4 | 1 | 0.115 |
| MS36 | LT10 peptide | 3 X | 4 X | BT-8 | 9 | 0.13 |
|  |  | 7 X | 8 X | BS-7 | 7 | 0.277 |
| MS37 | LT10 peptide | 3 X | 4 X | BT-8 | 9 | 0.13 |
|  |  | 7 X | 8 X | BS-8 | 2 | 0.128 |
| MS38 | LT10 peptide | 3 X | 4 X | BT-8 | 9 | 0.13 |
|  |  | 7 X | 8 X | BS-9 | 8 | 0.17 |
| MS39 | LT10 peptide | 3 X | 4 X | BT-8 | 9 | 0.13 |
|  |  | 7 X | 8 X | PdPP | 1 | 0.379 |
| MS40 | LT10 peptide | 3 X | 4 X | BT-8 | 9 | 0.13 |
|  |  | 7 X | 8 X | 1W3C_B | 3 | 0.165 |
| MS41 | LT10 peptide | 3 X | 4 X | BT-8 | 9 | 0.13 |
|  |  | 7 X | 8 X | 2AIG_I | 10 | 0.118 |
| MS42 | LT10 peptide | 3 X | 4 X | BT-8 | 9 | 0.13 |
|  |  | 7 X | 8 X | 3AIG_I | 6 | 0.117 |
| MS43 | LT10 peptide | 3 X | 4 X | BT-8 | 9 | 0.13 |
|  |  | 7 X | 8 X | M-1 | 4 | 0.06 |
| MS44 | LT10 peptide | 3 X | 4 X | BT-8 | 9 | 0.13 |
|  |  | 7 X | 8 X | M-2 | 3 | 0.086 |
| MS45 | LT10 peptide | 3 X | 4 X | BS-12 | 3 | 0.14 |
|  |  | 7 X | 8 X | BS-3 | 4 | 0.073 |
| MS46 | LT10 peptide | 3 X | 4 X | BS-12 | 3 | 0.14 |
|  |  | 7 X | 8 X | BS-4 | 1 | 0.115 |
| MS47 | LT10 peptide | 3 X | 4 X | BS-12 | 3 | 0.14 |
|  |  | 7 X | 8 X | BS-7 | 7 | 0.277 |
| MS48 | LT10 peptide | 3 X | 4 X | BS-12 | 3 | 0.14 |
|  |  | 7 X | 8 X | BS-8 | 2 | 0.128 |
| MS49 | LT10 peptide | 3 X | 4 X | BS-12 | 3 | 0.14 |
|  |  | 7 X | 8 X | BS-9 | 8 | 0.17 |
| MS50 | LT10 peptide | 3 X | 4 X | BS-12 | 3 | 0.14 |
|  |  | 7 X | 8 X | PdPP | 1 | 0.379 |
| MS51 | LT10 peptide | 3 X | 4 X | BS-12 | 3 | 0.14 |
|  |  | 7 X | 8 X | 1W3C_B | 3 | 0.165 |
| MS52 | LT10 peptide | 3 X | 4 X | BS-12 | 3 | 0.14 |
|  |  | 7 X | 8 X | 2AIG_I | 10 | 0.118 |
| MS53 | LT10 peptide | 3 X | 4 X | BS-12 | 3 | 0.14 |
|  |  | 7 X | 8 X | 3AIG_I | 6 | 0.117 |
| MS54 | LT10 peptide | 3 X | 4 X | BS-12 | 3 | 0.14 |
|  |  | 7 X | 8 X | M-1 | 4 | 0.06 |
| MS55 | LT10 peptide | 3 X | 4 X | BS-12 | 3 | 0.14 |
|  |  | 7 X | 8 X | M-2 | 3 | 0.086 |
| MS56 | LT10 peptide | 3 X | 4 X | BS-13 | 3 | 0.062 |
|  |  | 7 X | 8 X | BS-3 | 4 | 0.073 |
| MS57 | LT10 peptide | 3 X | 4 X | BS-13 | 3 | 0.062 |
|  |  | 7 X | 8 X | BS-4 | 1 | 0.115 |
| MS58 | LT10 peptide | 3 X | 4 X | BS-13 | 3 | 0.062 |
|  |  | 7 X | 8 X | BS-7 | 7 | 0.277 |
| MS59 | LT10 peptide | 3 X | 4 X | BS-13 | 3 | 0.062 |
|  |  | 7 X | 8 X | BS-8 | 2 | 0.128 |
| MS60 | LT10 peptide | 3 X | 4 X | BS-13 | 3 | 0.062 |
|  |  | 7 X | 8 X | BS-9 | 8 | 0.17 |
| MS61 | LT10 peptide | 3 X | 4 X | BS-13 | 3 | 0.062 |
|  |  | 7 X | 8 X | PdPP | 1 | 0.379 |
| MS62 | LT10 peptide | 3 X | 4 X | BS-13 | 3 | 0.062 |
|  |  | 7 X | 8 X | 1W3C_B | 3 | 0.165 |
| MS63 | LT10 peptide | 3 X | 4 X | BS-13 | 3 | 0.062 |
|  |  | 7 X | 8 X | 2AIG_I | 10 | 0.118 |
| MS64 | LT10 peptide | 3 X | 4 X | BS-13 | 3 | 0.062 |
|  |  | 7 X | 8 X | 3AIG_I | 6 | 0.117 |
| MS65 | LT10 peptide | 3 X | 4 X | BS-13 | 3 | 0.062 |
|  |  | 7 X | 8 X | M-1 | 4 | 0.06 |
| MS66 | LT10 peptide | 3 X | 4 X | BS-13 | 3 | 0.062 |
|  |  | 7 X | 8 X | M-2 | 3 | 0.086 |
| MS67 | LT10 peptide | 3 X | 4 X | 1A61_R | 10 | 0.115 |
|  |  | 7 X | 8 X | BS-3 | 4 | 0.073 |
| MS68 | LT10 peptide | 3 X | 4 X | 1A61_R | 10 | 0.115 |
|  |  | 7 X | 8 X | BS-4 | 1 | 0.115 |
| MS69 | LT10 peptide | 3 X | 4 X | 1A61_R | 10 | 0.115 |
|  |  | 7 X | 8 X | BS-7 | 7 | 0.277 |
| MS70 | LT10 peptide | 3 X | 4 X | 1A61_R | 10 | 0.115 |
|  |  | 7 X | 8 X | BS-8 | 2 | 0.128 |
| MS71 | LT10 peptide | 3 X | 4 X | 1A61_R | 10 | 0.115 |
|  |  | 7 X | 8 X | BS-9 | 8 | 0.17 |
| MS72 | LT10 peptide | 3 X | 4 X | 1A61_R | 10 | 0.115 |
|  |  | 7 X | 8 X | PdPP | 1 | 0.379 |
| MS73 | LT10 peptide | 3 X | 4 X | 1A61_R | 10 | 0.115 |
|  |  | 7 X | 8 X | 1W3C_B | 3 | 0.165 |
| MS74 | LT10 peptide | 3 X | 4 X | 1A61_R | 10 | 0.115 |
|  |  | 7 X | 8 X | 2AIG_I | 10 | 0.118 |
| MS75 | LT10 peptide | 3 X | 4 X | 1A61_R | 10 | 0.115 |
|  |  | 7 X | 8 X | 3AIG_I | 6 | 0.117 |
| MS76 | LT10 peptide | 3 X | 4 X | 1A61_R | 10 | 0.115 |
|  |  | 7 X | 8 X | M-1 | 4 | 0.06 |
| MS77 | LT10 peptide | 3 X | 4 X | 1A61_R | 10 | 0.115 |
|  |  | 7 X | 8 X | M-2 | 3 | 0.086 |
| MS78 | LT10 peptide | 3 X | 4 X | 1W3C_B | 5 | 0.138 |
|  |  | 7 X | 8 X | BS-3 | 4 | 0.073 |
| MS79 | LT10 peptide | 3 X | 4 X | 1W3C_B | 5 | 0.138 |
|  |  | 7 X | 8 X | BS-4 | 1 | 0.115 |
| MS80 | LT10 peptide | 3 X | 4 X | 1W3C_B | 5 | 0.138 |
|  |  | 7 X | 8 X | BS-7 | 7 | 0.277 |
| MS81 | LT10 peptide | 3 X | 4 X | 1W3C_B | 5 | 0.138 |
|  |  | 7 X | 8 X | BS-8 | 2 | 0.128 |
| MS82 | LT10 peptide | 3 X | 4 X | 1W3C_B | 5 | 0.138 |
|  |  | 7 X | 8 X | BS-9 | 8 | 0.17 |
| MS83 | LT10 peptide | 3 X | 4 X | 1W3C_B | 5 | 0.138 |
|  |  | 7 X | 8 X | PdPP | 1 | 0.379 |
| MS84 | LT10 peptide | 3 X | 4 X | 1W3C_B | 5 | 0.138 |
|  |  | 7 X | 8 X | 1W3C_B | 3 | 0.165 |
| MS85 | LT10 peptide | 3 X | 4 X | 1W3C_B | 5 | 0.138 |
|  |  | 7 X | 8 X | 2AIG_I | 10 | 0.118 |
| MS86 | LT10 peptide | 3 X | 4 X | 1W3C_B | 5 | 0.138 |
|  |  | 7 X | 8 X | 3AIG_I | 6 | 0.117 |
| MS87 | LT10 peptide | 3 X | 4 X | 1W3C_B | 5 | 0.138 |
|  |  | 7 X | 8 X | M-1 | 4 | 0.06 |
| MS88 | LT10 peptide | 3 X | 4 X | 1W3C_B | 5 | 0.138 |
|  |  | 7 X | 8 X | M-2 | 3 | 0.086 |
| MS89 | LT10 peptide | 3 X | 4 X | 2AIG_I | 5 | 0.154 |
|  |  | 7 X | 8 X | BS-3 | 4 | 0.073 |
| MS90 | LT10 peptide | 3 X | 4 X | 2AIG_I | 5 | 0.154 |
|  |  | 7 X | 8 X | BS-4 | 1 | 0.115 |
| MS91 | LT10 peptide | 3 X | 4 X | 2AIG_I | 5 | 0.154 |
|  |  | 7 X | 8 X | BS-7 | 7 | 0.277 |
| MS92 | LT10 peptide | 3 X | 4 X | 2AIG_I | 5 | 0.154 |
|  |  | 7 X | 8 X | BS-8 | 2 | 0.128 |
| MS93 | LT10 peptide | 3 X | 4 X | 2AIG_I | 5 | 0.154 |
|  |  | 7 X | 8 X | BS-9 | 8 | 0.17 |
| MS94 | LT10 peptide | 3 X | 4 X | 2AIG_I | 5 | 0.154 |
|  |  | 7 X | 8 X | PdPP | 1 | 0.379 |
| MS95 | LT10 peptide | 3 X | 4 X | 2AIG_I | 5 | 0.154 |
|  |  | 7 X | 8 X | 1W3C_B | 3 | 0.165 |
| MS96 | LT10 peptide | 3 X | 4 X | 2AIG_I | 5 | 0.154 |
|  |  | 7 X | 8 X | 2AIG_I | 10 | 0.118 |
| MS97 | LT10 peptide | 3 X | 4 X | 2AIG_I | 5 | 0.154 |
|  |  | 7 X | 8 X | 3AIG_I | 6 | 0.117 |
| MS98 | LT10 peptide | 3 X | 4 X | 2AIG_I | 5 | 0.154 |
|  |  | 7 X | 8 X | M-1 | 4 | 0.06 |
| MS99 | LT10 peptide | 3 X | 4 X | 2AIG_I | 5 | 0.154 |
|  |  | 7 X | 8 X | M-2 | 3 | 0.086 |
