# Supplementary material for: An In Silico Insight into Novel Therapeutic Interaction of LTNF Peptide-LT10 and Design of Structure Based Peptidomimetics for Putative Anti-Diabetic Activity
Source: PLoS One. 2015 Mar 27;10(3):e0121860. doi: 10.1371/journal.pone.0121860 (PMC4376886; doi:10.1371/journal.pone.0121860)
Supplement: S4 Table — (DOCX) [file pone.0121860.s008.docx]

**S4 Table. Subset of Type 1 peptidomimetics – 5mer and 6mer.**

| **Sr. no.** | **Protein template** | **stem_N** | **stem_C** | **mimetic** | **conformation** | **RMSD (Å)** |
| --- | --- | --- | --- | --- | --- | --- |
| 1 | LT5 peptide  ^1^**LKAMD**^5^ | 3 X | 4 X | AH-5 | 1 | 0.166 |
| 2 |  | 3 X | 4 X | BT-3 | 3 | 0.213 |
| 3 |  | 3 X | 4 X | BT-7 | 2 | 0.164 |
| 4 |  | 3 X | 4 X | BT-8 | 9 | 0.13 |
| 5 |  | 3 X | 4 X | BS-12 | 3 | 0.14 |
| 6 |  | 3 X | 4 X | BS-13 | 3 | 0.062 |
| 8 |  | 3 X | 4 X | 1A61_R | 10 | 0.115 |
| 9 |  | 3 X | 4 X | 1W3C_B | 5 | 0.138 |
| 10 |  | 3 X | 4 X | 2AIG_I | 5 | 0.154 |
| 1 | LT6 peptide  ^1^**LKAMDP**^6^ | 3 X | 4 X | AH-5 | 1 | 0.166 |
| 2 |  | 3 X | 4 X | BT-3 | 3 | 0.213 |
| 3 |  | 3 X | 4 X | BT-7 | 2 | 0.164 |
| 4 |  | 3 X | 4 X | BT-8 | 9 | 0.13 |
| 5 |  | 3 X | 4 X | BS-12 | 3 | 0.14 |
| 6 |  | 3 X | 4 X | BS-13 | 3 | 0.062 |
| 8 |  | 3 X | 4 X | 1A61_R | 10 | 0.115 |
| 9 |  | 3 X | 4 X | 1W3C_B | 5 | 0.138 |
| 10 |  | 3 X | 4 X | 2AIG_I | 5 | 0.154 |
