# Supplementary material for: An In Silico Insight into Novel Therapeutic Interaction of LTNF Peptide-LT10 and Design of Structure Based Peptidomimetics for Putative Anti-Diabetic Activity
Source: PLoS One. 2015 Mar 27;10(3):e0121860. doi: 10.1371/journal.pone.0121860 (PMC4376886; doi:10.1371/journal.pone.0121860)
Supplement: S5 Table — (DOCX) [file pone.0121860.s009.docx]

**S5 Table. Chemical details of best peptidomimetics inhibitors of IDE designed from LT10 peptide.**

| **Type** | **Peptidomimetic** | **SMILES** | **IUPAC name** |
| --- | --- | --- | --- |
| Type 1 | LKAMDP(**BS-8**)PL | O=C(NC(C=O)CC(C)C)C3N(C(O)(O)CN2C(=O)C(NC(=O)C1N(C(=O)C(NC(=O)C(NC(=O)C(NC(=O)C(NC(=O)C([NH3+])CC(C)C)CCCC[NH3+])C)CCSC)CC([O-])=O)CCC1)CCC2C)CCC3 | (3S)-3-[(2S)-2-[(2S)-2-[(2S)-6-azaniumyl-2-[(2S)-2-azaniumyl-4-methylpentanamido]hexanamido]propanamido]-4-(methylsulfanyl)butanamido]-4-[(2S)-2-{[(6R)-1-{2,2-dihydroxy-2-[(2S)-2-{[(2S)-4-methyl-1-oxopentan-2-yl]carbamoyl}pyrrolidin-1-yl]ethyl}-6-methyl-2-oxopiperidin-3-yl]carbamoyl}pyrrolidin-1-yl]-4-oxobutanoate |
| Type 1 | LKAMDP(**M-2**)PL | O=C(NC(C=O)CC(C)C)C3N(C(O)(O)CN2C(=O)C(CC(=O)C1N(C(=O)C(NC(=O)C(NC(=O)C(NC(=O)C(NC(=O)C([NH3+])CC(C)C)CCCC[NH3+])C)CCSC)CC([O-])=O)CCC1)CCC2)CCC3 | (3S)-3-[(2S)-2-[(2S)-2-[(2S)-6-azaniumyl-2-[(2S)-2-azaniumyl-4-methylpentanamido]hexanamido]propanamido]-4-(methylsulfanyl)butanamido]-4-[(2S)-2-{2-[(3S)-1-{2,2-dihydroxy-2-[(2S)-2-{[(2S)-4-methyl-1-oxopentan-2-yl]carbamoyl}pyrrolidin-1-yl]ethyl}-2-oxopiperidin-3-yl]acetyl}pyrrolidin-1-yl]-4-oxobutanoate |
| Type 1 | LK(**BT-8**)DPTPPL | O=C(N1C(C(=O)NC(C=O)CC(C)C)CCC1)C4N(C(=O)C(NC(=O)C3N(C(=O)C(NC(O)(O)C2NC(=O)C(NC(=O)C(NC(=O)C([NH3+])CC(C)C)CCCC[NH3+])CCNC(=O)CC2)CC([O-])=O)CCC3)C(O)C)CCC4 | (3S)-3-({[(2R,9S)-9-[(2S)-6-azaniumyl-2-[(2S)-2-azaniumyl-4-methylpentanamido]hexanamido]-5,10-dioxo-1,6-diazecan-2-yl]dihydroxymethyl}amino)-4-[(2S)-2-{[(2S,3R)-3-hydroxy-1-[(2S)-2-[(2S)-2-{[(2S)-4-methyl-1-oxopentan-2-yl]carbamoyl}pyrrolidine-1-carbonyl]pyrrolidin-1-yl]-1-oxobutan-2-yl]carbamoyl}pyrrolidin-1-yl]-4-oxobutanoate |
| Type 2 | LK(**BT-3**)DP(**BS-3**)PL | O=CC(NC(=O)C1N(CCC1)C(O)(O)C3CCCN2C(O)C(C(O)N23)(NC(=O)C6N(C(=O)C(NC(O)(O)C4N5CC(NC(=O)C(NC(=O)C([NH3+])CC(C)C)CCCC[NH3+])CCC5SC4)CC([O-])=O)CCC6)C)CC(C)C | (3S)-3-({[(3R,6S,8aS)-6-[(2S)-6-azaniumyl-2-[(2S)-2-azaniumyl-4-methylpentanamido]hexanamido]-hexahydro-2H-[1,3]thiazolo[3,2-a]pyridin-3-yl]dihydroxymethyl}amino)-4-[(2S)-2-{[(2R,5S)-5-{dihydroxy[(2S)-2-{[(2S)-4-methyl-1-oxopentan-2-yl]carbamoyl}pyrrolidin-1-yl]methyl}-1,3-dihydroxy-2-methyl-hexahydro-1H-pyrazolidino[1,2-a]pyridazin-2-yl]carbamoyl}pyrrolidin-1-yl]-4-oxobutanoate |
| Type 2 | LK(**AH-5**)DP(**BS-9**)PL | O=C(NC(C=O)CC(C)C)C5N(C(O)(O)CN4C(O)C(NC(=O)C3N(C(=O)C(NC(O)(O)C2(C1CC(C)C(C(=O)C(NC(=O)C([NH3+])CC(C)C)CCCC[NH3+])CC1CC2)C)CC([O-])=O)CCC3)CCCC4)CCC5 | (3S)-3-({[(1R,5R)-5-[(2S)-6-azaniumyl-2-[(2S)-2-azaniumyl-4-methylpentanamido]hexanoyl]-1,6-dimethyl-octahydro-1H-inden-1-yl]dihydroxymethyl}amino)-4-[(2S)-2-{[(3S)-1-{2,2-dihydroxy-2-[(2S)-2-{[(2S)-4-methyl-1-oxopentan-2-yl]carbamoyl}pyrrolidin-1-yl]ethyl}-2-hydroxyazepan-3-yl]carbamoyl}pyrrolidin-1-yl]-4-oxobutanoate |
| Type 2 | LK(**BT-8**)DP(**BS-7**)PL | O=C(NC(C=O)CC(C)C)C4N(C(O)(O)CN3C(O)C(NC(=O)C2N(C(=O)C(NC(O)(O)C1NC(O)C(NC(=O)C(NC(=O)C([NH3+])CC(C)C)CCCC[NH3+])CCNC(O)CC1)CC([O-])=O)CCC2)CCC3)CCC4 | (3S)-3-({[(2R,9S)-9-[(2S)-6-azaniumyl-2-[(2S)-2-azaniumyl-4-methylpentanamido]hexanamido]-5,10-dihydroxy-1,6-diazecan-2-yl]dihydroxymethyl}amino)-4-[(2S)-2-{[(3S)-1-{2,2-dihydroxy-2-[(2S)-2-{[(2S)-4-methyl-1-oxopentan-2-yl]carbamoyl}pyrrolidin-1-yl]ethyl}-2-hydroxypiperidin-3-yl]carbamoyl}pyrrolidin-1-yl]-4-oxobutanoate |
| Subset 5mer | LK(**BT-7**)D | [O]C(=O)CC(C=O)NC(O)(O)C1NC(O)C(NC(=O)C(NC(=O)C([NH3+])CC(C)C)CCCC[NH3+])CCCCCC1 | (3S)-3-({[(2R,9S)-9-[(2S)-6-azaniumyl-2-[(2S)-2-azaniumyl-4-methylpentanamido]hexanamido]-10-hydroxyazecan-2-yl]dihydroxymethyl}amino)-4-oxobutanoate |
| Subset 6mer | LK(**BT-3**)DP | O=CC3N(C(=O)C(NC(O)(O)C1N2CC(NC(=O)C(NC(=O)C([NH3+])CC(C)C)CCCC[NH3+])CCC2SC1)CC([O-])=O)CCC3 | (3S)-3-({[(3R,6S,8aS)-6-[(2S)-6-azaniumyl-2-[(2S)-2-azaniumyl-4-methylpentanamido]hexanamido]-hexahydro-2H-[1,3]thiazolo[3,2-a]pyridin-3-yl]dihydroxymethyl}amino)-4-[(2S)-2-formylpyrrolidin-1-yl]-4-oxobutanoate |
| Subset 6mer | LK(**BT-8**)DP | O=C(N1C(C(=O)NC(C=O)CC(C)C)CCC1)C4N(C(=O)C(NC(=O)C3N(C(=O)C(NC(O)(O)C2NC(=O)C(NC(=O)C(NC(=O)C([NH3+])CC(C)C)CCCC[NH3+])CCNC(=O)CC2)CC([O-])=O)CCC3)C(O)C)CCC4 | (3S)-3-({[(2R,9S)-9-[(2S)-6-azaniumyl-2-[(2S)-2-azaniumyl-4-methylpentanamido]hexanamido]-5,10-dioxo-1,6-diazecan-2-yl]dihydroxymethyl}amino)-4-[(2S)-2-{[(2S,3R)-3-hydroxy-1-[(2S)-2-[(2S)-2-{[(2S)-4-methyl-1-oxopentan-2-yl]carbamoyl}pyrrolidine-1-carbonyl]pyrrolidin-1-yl]-1-oxobutan-2-yl]carbamoyl}pyrrolidin-1-yl]-4-oxobutanoate |
